# An Argon-Ion-Induced Pale Green Mutant of *Arabidopsis* Exhibiting Rapid Disassembly of Mesophyll Chloroplast Grana

**DOI:** 10.3390/plants10050848

**Published:** 2021-04-22

**Authors:** Alvin Sanjaya, Yusuke Kazama, Kotaro Ishii, Ryohsuke Muramatsu, Kengo Kanamaru, Sumie Ohbu, Tomoko Abe, Makoto T. Fujiwara

**Affiliations:** 1 Faculty of Science and Technology, Sophia University, 7-1 Kioicho, Chiyoda, Tokyo 102-8554, Japan; alvin_sanjaya@protonmail.com (A.S.); ryohsuke67@outlook.com (R.M.); 2 RIKEN Nishina Center, 2-1 Hirosawa, Wako, Saitama 351-0198, Japan; kotaro@riken.jp (K.I.); ohbu@riken.jp (S.O.); tomoabe@riken.jp (T.A.); 3 Faculty of Bioscience and Biotechnology, Fukui Prefectural University, 4-1-1 Matsuoka-Kenjojima, Eiheiji, Yoshida, Fukui 910-1195, Japan; 4 Faculty of Agriculture, Kobe University, Nada, Kobe, Hyogo 657-8501, Japan; kng@kobe-u.ac.jp

**Keywords:** *Arabidopsis thaliana*, *EGY1*, leaf senescence, heavy-ion beam mutagenesis, plastid

## Abstract

Argon-ion beam is an effective mutagen capable of inducing a variety of mutation types. In this study, an argon ion-induced pale green mutant of *Arabidopsis thaliana* was isolated and characterized. The mutant, designated Ar50-33-pg1, exhibited moderate defects of growth and greening and exhibited rapid chlorosis in photosynthetic tissues. Fluorescence microscopy confirmed that mesophyll chloroplasts underwent substantial shrinkage during the chlorotic process. Genetic and whole-genome resequencing analyses revealed that Ar50-33-pg1 contained a large 940 kb deletion in chromosome V that encompassed more than 100 annotated genes, including 41 protein-coding genes such as *TYRAAt1*/*TyrA1*, *EGY1*, and *MBD12*. One of the deleted genes, *EGY1*, for a thylakoid membrane-localized metalloprotease, was the major contributory gene responsible for the pale mutant phenotype. Both an *egy1* mutant and F_1_ progeny of an Ar50-33-pg1 × *egy1* cross-exhibited chlorotic phenotypes similar to those of Ar50-33-pg1. Furthermore, ultrastructural analysis of mesophyll cells revealed that Ar50-33-pg1 and *egy1* initially developed wild type-like chloroplasts, but these were rapidly disassembled, resulting in thylakoid disorganization and fragmentation, as well as plastoglobule accumulation, as terminal phenotypes. Together, these data support the utility of heavy-ion mutagenesis for plant genetic analysis and highlight the importance of *EGY1* in the structural maintenance of grana in mesophyll chloroplasts.

## 1. Introduction

Chloroplasts belong to a class of double-envelope membrane-bound organelles—the plastids. Chloroplasts contain the thylakoid system for light absorption and energy production, and nucleoids for DNA replication and gene expression, as their major structural components [1,2,3]. Chloroplasts are indispensable for plant growth and development, acting as the site of carbon and nitrogen assimilation and as a synthesis site for amino acids, fatty acids, vitamins, and other cellular components. Compared with the cell nucleus and mitochondria, plastids undergo flexible morphological alterations that depend on the plant tissues and cells in which they reside. In this context, chloroplast development in photosynthetic tissues is one of the most remarkable features of plastid differentiation. During leaf development of the model plant *Arabidopsis thaliana*, proplastids, a primitive plastid form (~1 µm in diameter) with rudimentary internal membranes in meristematic tissues, actively proliferate and expand several hundred-fold, a process that accompanies massive development of thylakoids. This results in the formation of typically 6 µm (usually 3–10 µm in diameter) chloroplasts in mature mesophyll cells [2,3]. Upon leaf aging, the light-absorbing chlorophyll pigments undergo catabolism and transform into non-green derivatives, but the relative intracellular chloroplast number is maintained until later stages of leaf senescence [4,5]. Additionally, the leaf senescence program exhibits progressive destruction of the thylakoids and the concomitant accumulation of lipid-enriched plastoglobule structures, which are generated as a consequence of thylakoid degradation [6,7]. The extent of chloroplast biogenesis and degeneration in leaves is reflected in leaf chlorophyll levels, which can be used as an indicator of leaf developmental stage and physiological state to understand chloroplast differentiation, proliferation, and degeneration.

The chloroplast proteome is composed of several thousand different proteins [8]. In support of an endosymbiotic theory, modern-day chloroplasts retained a minimal set of cyanobacterial genes in their own genomes, while a group of putative ancestral prokaryotic genes transferred to the host nucleus during plant evolution [9,10]. The structure and function of modern chloroplasts are under the strict control of the cell nucleus. Approximately 90% of chloroplast proteins are nuclear-encoded, with the remaining 10% being encoded by the chloroplast. Mutagenesis technologies, combined with molecular and cell biological methodologies, are powerful tools for understanding the molecular mechanisms of chloroplast development in plants. Chemical methods (e.g., ethylmethane sulfonate (EMS) treatment), physical methods (e.g., ultraviolet light, X-ray, gamma-ray, and fast neutron irradiations), and biological methods (e.g., T-DNA and transposon insertions) are widely used in several plant experimental systems (e.g., the Chloroplast 2010 project [11,12]). In *A. thaliana*, there are approximately 26,000 protein-coding genes in the 135 Mb nuclear genome, and numerous mutants defective in functions such as chloroplast transcription, translation, division, protein import, protein quality control, photosynthesis, and chloroplast-nucleus signaling have been isolated [8,13,14,15,16]. One such chloroplast biogenesis mutant, *egy1*, was identified through screening of EMS-induced populations [17]. *EGY1* encodes a thylakoid membrane-spanning metalloprotease whose precise functions remain to be elucidated [17,18,19,20,21,22]. Chloroplast biogenesis mutants are often categorized into one of several types according to their appearance. These types include *albino*, for complete or extreme loss of leaf pigments; *pale*; *pale green*; *yellow green*; *lutescent*, for partial loss of chlorophylls in leaves; *virescent*, for mutants with a pale leaf phenotype with late greening characteristics; *variegation*, for non-uniform, sometimes structurally caused leaf coloration [23,24]. In many cases, however, the molecular backgrounds underlying leaf coloration phenotypes remain unclear.

Heavy-ion beam is an effective mutagen that causes DNA lesions in target tissues. Accelerated heavy ions can produce double-stranded DNA breaks, which then undergo repair through non-homologous end joining (NHEJ) pathways that often involve microhomology-dependent repair. Several mutation types have been reported in heavy-ion mutagenesis research, including base substitutions, small (single-to-several nucleotide) deletions, inversions, large deletions, and chromosomal rearrangements. In the last decade, our research has investigated the effects of nuclide species, linear energy transfer (LET) values, dose amount, and target plant conditions on the structure of DNA mutations in targeted *A. thaliana* tissues [25,26,27,28,29,30,31]. Among the four nuclides tested (^12^C^6+^, ^20^Ne^10+^, ^40^Ar^17+^, and ^56^Fe^24+^), argon-ion beams were attractive, particularly in comparison with carbon-ion beams, as they produced a broad spectrum of mutations and could induce complex and large-scale mutations at random genomic sites [27,30,31]. Heavy-ion mutagenesis is an established technique in applied plant science, such as in crop and ornamental plant breeding [32,33], and also has strong potential for use in fundamental plant science. This motivated our exploration of heavy-ion induced *A. thaliana* mutants with intriguing chloroplast phenotypes.

In this study, a chloroplast-related mutant of *A. thaliana* was identified from a screen using argon-ion irradiation. The mutant, Ar50-33-pg1, contained a recessive mutation that comprised a 940 kb deletion in chromosome V of the nuclear genome. Several genes were lost in the Ar50-33-pg1 deletion, one of which, *EGY1* [17], was a plausible causative gene for the pale phenotype. Microscopic analysis revealed that *EGY1* had previously unknown functions in mesophyll chloroplasts. The results of this investigation and the facility of the Ar50-33-pg1 mutant in plant research are discussed.

## 2. Results

### 2.1. Isolation of Ar50-33-pg1, a Pale Green Mutant by Argon-Ion Irradiation

**Figure 1:**
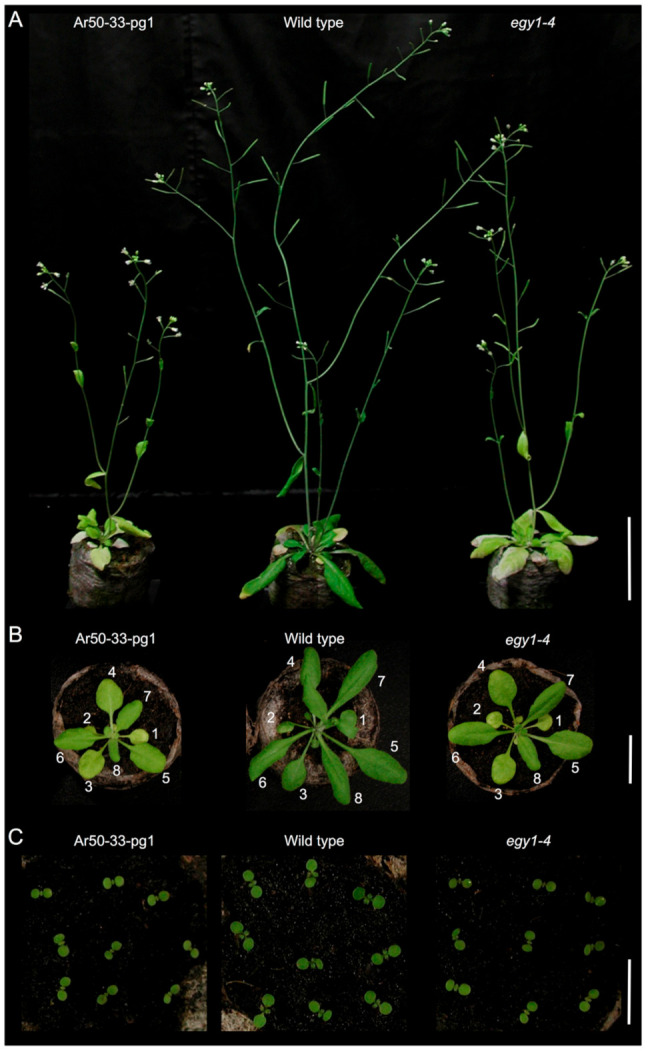
Growth and shoot morphology of wild type and mutant plants. (**A**) Five-week-old Ar50-33-pg1, wild type, and *egy1-4* plants. (**B**) Three-week-old plants. (**C**) One-week-old plants. Numbers in (**B**) indicate the order of leaf emergence. Bar = 5 cm (**A**), 2 cm (**B**), and 1 cm (**C**).

Dry seeds of *A. thaliana* Col were exposed to argon-ions accelerated at the Radioactive Isotope Beam Factory (RIBF), RIKEN [27,31]. After sowing, M_1_ plants were grown and selfed to amplify the M_2_ and M_3_ seeds. During plant cultivation, a candidate mutant with a stable pale green phenotype at the seedling and flowering stages was macroscopically screened (Figure 1 and Figure S1) and designated Ar50-33-pg1. To determine the genetic cause of the pale mutant phenotype, Ar50-33-pg1 was backcrossed with Col in both directions (Table 1). All F_1_ seedlings were normal green and F_2_ seedlings segregated in a 3:1 ratio of normal green-to-pale green phenotypes, indicating Mendelian inheritance. Ar50-33-pg1, therefore, carried a single, recessive mutation in the nuclear genome.

**Figure 2:**
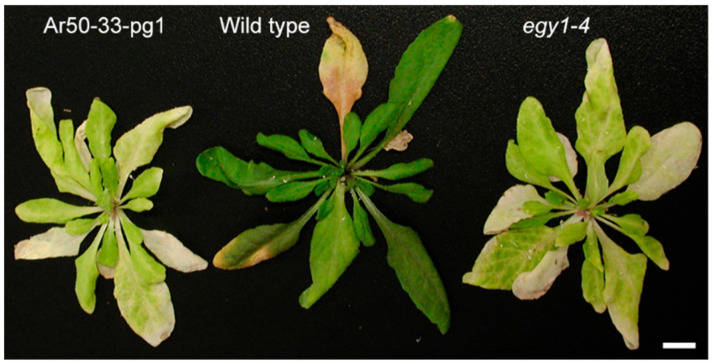
Rosette morphology of 6-week-old wild type and mutant plants. Stems were removed just prior to image capture. Bar = 1 cm.

The gross morphology of Ar50-33-pg1 was examined under a long-day condition (Figure 1). In the flowering stage [34], Ar50-33-pg1 plants exhibited relatively retarded growth and small leaves compared with wild type (Figure 1A). Ar50-33-pg1 leaves were yellowish to whitish green, which contrasted with the dark green leaves of wild type. Despite the apparent degreening events, the mutant leaves appeared to maintain viability to a wild type level over the cultivation period (Figure 2), indicating that the Ar50-33-pg1 pale leaf phenotype was due to chlorosis rather than senescence [35]. The pale leaves of Ar50-33-pg1 were less apparent in younger plant stages. Compared with wild type leaves, 3-week-old mutant leaves were yellowish green with a mildly reduced size (Figure 1B), whereas 1-week-old mutant seedlings were distinguishable from wild type seedlings only by their slightly paler cotyledons (Figure 1C and Figure S1). When compared with a pale green (virescent) mutant, *sig2* (*sig2-2* allele; [36,37]), in which chloroplast development was severely impaired in emerging leaves as a result of chloroplast tRNA deficiency [38,39,40], expansion and greening of new Ar50-33-pg1 leaves appeared relatively normal (Figure S1). However, in contrast to the delayed greening of *sig2-2* leaves [41], older leaves were considerably paler than younger leaves in Ar50-33-pg1 seedlings (Figure 1B). Ar50-33-pg1 seedlings produced 8–10 rosette leaves before bolting, and leaf age-dependent chlorosis was observed throughout plant growth (Figure 1 and Figure 2), indicating that Ar50-33-pg1 was a moderate chloroplast biogenesis-related mutant with chloroplast defects that increased with leaf aging.

Next, the stem phenotype of Ar50-33-pg1 at the flowering stage was examined (Figure S2). Greening of the shoot apex and associated inflorescence regions was similar in Ar50-33-pg1 and wild type, but chlorosis was apparent in the basal region of Ar50-33-pg1 stems. As a result, the stem of the mutant produced a green-to-yellow gradient from the tip to the base along the axis. This visual phenotype was confirmed at the chlorophyll autofluorescence level by fluorescence stereomicroscopy (Figure S2B). Thus, Ar50-33-pg1 stems also exhibited increasing chlorosis with increasing tissue age. Ar50-33-pg1 also exhibited several morphological abnormalities in reproductive tissues. First, due to the growth delay of Ar50-33-pg1 seedlings, bolting initiation was delayed and Ar50-33-pg1 displayed a late flowering phenotype (Figure 1A and Figure S3A). Days to first flowering (open flower formation) in Ar50-33-pg1 was 29.8 ± 2.0, compared with 23.5 ± 1.2 in wild type (*n* = 10). Ar50-33-pg1 also exhibited increased flower longevity due to a delay of flower senescence (Figure S3B). Ar50-33-pg1 inflorescences produced 7–8 open flowers with intact floral organs, sepals, petals, stamens, and pistils. Ar50-33-pg1 stamens exhibited anther dehiscence (Figure S3C), but fewer seeds were set in the mutant than in wild type (Figure S3C; data not shown). At the early flowering stage, siliques were shorter in the mutant than in wild type, and their surface had a pale appearance (Figure S3D). The short silique and reduced seed production phenotypes were gradually recovered at the later flowering stages (data not shown).

### 2.2. Chlorosis of Ar50-33-pg1 Leaves Is Associated with Impaired Chloroplast Morphology in Mesophyll Cells

**Figure 3:**
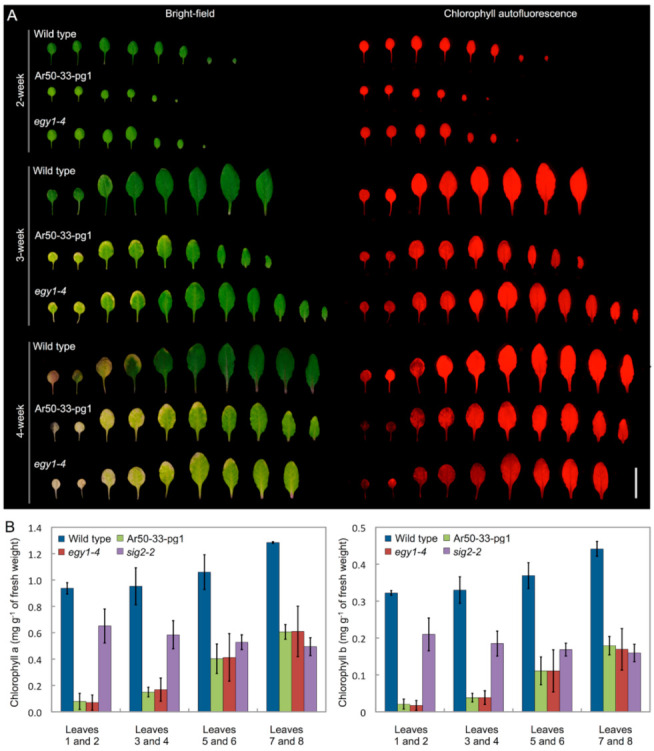
Leaf phenotypes of wild type and mutant plants. (**A**) Bright-field and chlorophyll autofluorescence images of detached leaves from 2-, 3- and 4-week-old wild type, Ar50-33-pg1, and *egy1-4* plants (from left to right: oldest to youngest leaves). Bar = 2 cm. (**B**) Measurement of chlorophyll *a* and chlorophyll *b* in wild type, Ar50-33-pg1, *egy1-4*, and *sig2-2* leaves. Three independent experiments were performed using 4-week-old leaves (error bars represent the SD).

To further characterize the pale phenotype of Ar50-33-pg1, leaf formation was compared in wild type and mutant seedlings using fluorescence stereomicroscopy (Figure 3A). Under a long-day condition, both mutant and wild type seedlings produced eight or more rosette leaves before bolting. Relative leaf configurations and leaf expansions were comparable in the mutant and wild type. However, onset of chlorosis in Ar50-33-pg1 leaves occurred prior to full expansion, in contrast to wild type leaves, which exhibited senescence after maturation [4,42]. In wild type leaves, chlorosis typically began at the tip and the marginal regions in the tip half of leaves, subsequently spreading toward the inner leaf regions. In Ar50-33-pg1 leaves, chlorosis occurred not only at the tip but in a patchy pattern across the leaf blade. These leaf chlorotic processes were confirmed by the loss of chlorophyll autofluorescence signals as observed by fluorescence stereomicroscopy and by using *sig2-2* leaves (Figure S4). Therefore, chlorosis progression differed spatially between wild type and mutant leaves. 

Next, chlorophyll levels were assessed by spectrophotometry (Figure 3B). Chlorophyll was extracted from four pairs of seedling leaves using a dimethylformamide-based method [43]. Wild type leaves exhibited gradual reductions in chl *a* and chl *b* levels from younger (leaves 7 and 8) to older (leaves 1 and 2) leaves. Compared with wild type, chl *a* and chl *b* levels in Ar50-33-pg1 leaves were reduced by approximately half in the youngest samples (leaves 7 and 8) and exhibited substantial additional reductions as leaf age increased. Consequently, the differences in chl *a* and chl *b* levels between wild type and mutant leaves were larger in older leaves than younger leaves. These trends were generally consistent with the microscopy data. As a reference, chlorophyll contents were also assessed in leaves of *sig2-2*, a virescent mutant similar to *sig2-1* [39,41]. In *sig2-2*, chl *a* and chl *b* levels were lower than wild type levels, but were slightly higher in older leaves (leaves 1 and 2) than in younger leaves (leaves 7 and 8). These results suggested that elevated chlorophyll degradation, rather than insufficient chlorophyll synthesis, was responsible for the age-related pale phenotype of Ar50-33-pg1.

**Figure 4:**
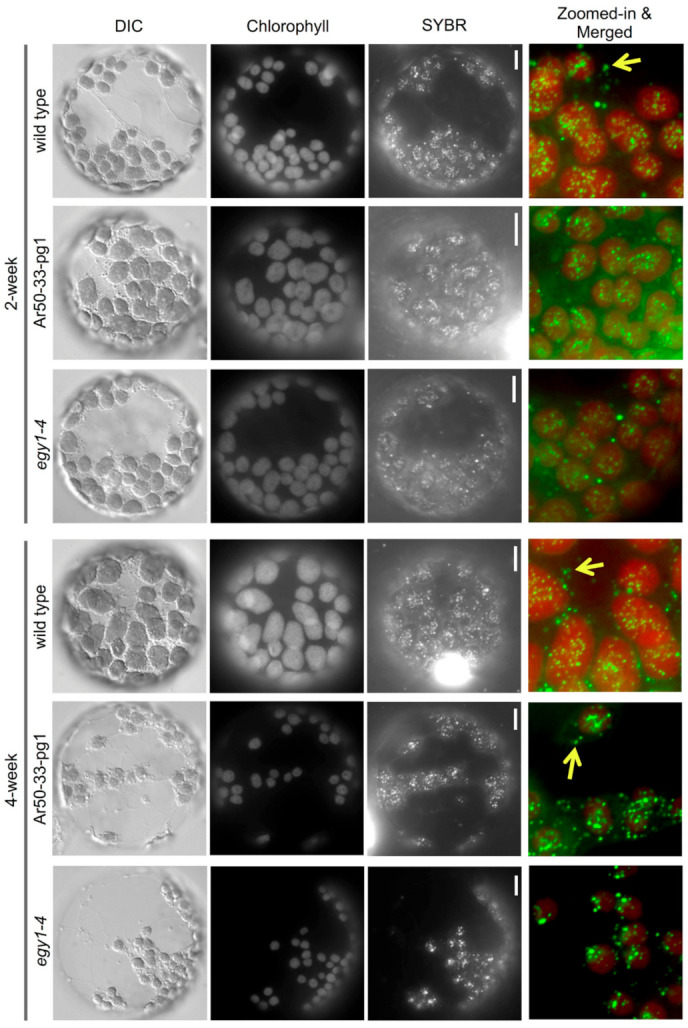
Fluorescence microscopy of leaf mesophyll protoplasts of wild type and mutant plants. Protoplasts from primary leaves of 2- and 4-week-old wild type, Ar50-33-pg1, and *egy1-4* seedlings were stained with SYBR Green. Images of DIC, chlorophyll autofluorescence, and SYBR Green fluorescence, and merged images of chlorophyll and SYBR Green fluorescence (chlorophyll in red and SYBR Green in green) are shown. Arrows indicate mitochondrial nucleoids. See also Figures S5–S7. Bar = 10 µm.

Next, microscopic characterization of leaf mesophyll cells was performed to investigate the cellular processes underlying Ar50-33-pg1 chlorosis. Plastid and mitochondrial DNAs in plants cells are organized in nucleoids [44,45]. The organization and distribution of chloroplast nucleoids can indicate chloroplast differentiation or degradation status [46,47] and, as chloroplast nucleoids are important in chloroplast gene expression, development, and phosphate supply, their analysis can provide valuable information regarding leaf physiology [48,49,50,51,52]. Therefore, we questioned whether chloroplast nucleoid formation might be affected in chlorosis-inducing mutants such as Ar50-33-pg1. Mesophyll protoplasts from relatively healthy primary leaves of 2- and 4-week-old wild type and mutant seedlings were prepared and stained with SYBR Green, which binds DNA (Figure 4, Figures S5 and S6). First, the length and shape of chloroplasts were affected. Wild type protoplasts contained chloroplasts with lengths of 4–10 µm (average, 6.4 µm; *n* = 30) in 2-week-old leaves and 5–12 µm (average, 8.2 µm; *n* = 30) in 4-week-old leaves. By contrast, mutant protoplasts contained chloroplasts of similar length in 2-week-old leaves (4–10 µm; average, 6.4 µm; *n* = 30) but shorter chloroplasts (3–9 µm; average, 5.2 µm; *n* = 30) in 4-week-old leaves. Moreover, the chloroplast shrinkage in older mutant leaves was accompanied by a uniform rounding of chloroplast shape, although no conspicuous alteration of the overall number of chloroplasts was observed (Figure 4, Figures S5 and S6). Second, chlorophyll accumulation differed between wild type and mutant chloroplasts. Wild type chloroplasts continued to accumulate chlorophylls during leaf expansion, and healthy mesophyll cell chloroplasts from 4-week-old leaves maintained a higher level of chlorophylls than in 2-week-old leaves. By contrast, Ar50-33-pg1 chloroplasts had lower chlorophyll fluorescence than wild type chloroplasts in 2-week-old leaves, and chlorophyll levels decreased further in the 4-week-old mutant samples (Figure 4, Figures S5 and S7). Chlorophyll fluorescence signal intensity was uniform in wild type and mutant chloroplasts, with the exception of heterogeneity observed in some mutant chloroplasts from 4-week-old leaves (see Figure S7). These observations suggested that the marked reduction in both chloroplast size and chlorophyll levels in mutant mesophyll cells might be responsible for the rapid chlorosis observed in Ar50-33-pg1 leaves. Finally, nucleoid distribution was unaffected in mutant chloroplasts. Individual nucleoids contain multiple DNA copies, and these can be detected within protoplasts using fluorescent dyes such DAPI [48,51] and, as in this study, SYBR Green. Mesophyll cells from 2- and 4-week-old leaves exhibited punctate chloroplast nucleoid distribution, and mitochondrial nucleoids were also stained (Figure 4, arrows). Chloroplast and mitochondrial nucleoid distribution did not noticeably differ between wild type and mutant mesophyll cells, despite considerable chloroplast shrinkage in Ar50-33-pg1 (Figure 4 and Figures S5–S7).

### 2.3. Identification of a Large Deletion Mutation in Ar50-33-pg1

**Figure 5:**
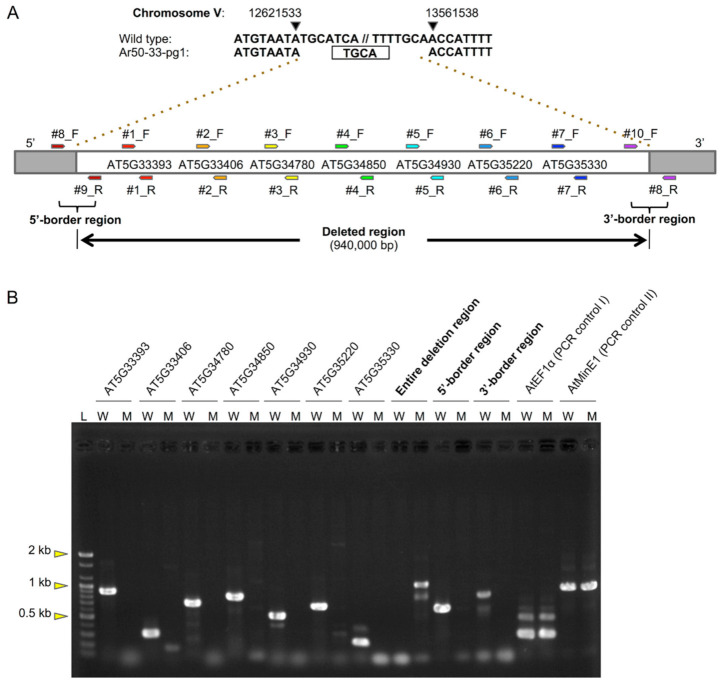
Large chromosomal deletion in Ar50-33-pg1 and genomic PCR analysis. (**A**) Schematic diagram of the deleted region on Ar50-33-pg1 chromosome V. Box denotes a microhomology sequence. Relative positions of genes and PCR primers are indicated. (**B**) Agarose gel electrophoresis of PCR products. L: DNA ladder marker, W: wild type, M: Ar50-33-pg1. Full details of deleted genes, PCR primers, and PCR products are in Table 2, Tables S1 and S2.

To identify the mutation responsible for the Ar50-33-pg1 pale phenotype, an M_3_ line was backcrossed with Col, and its F_2_ offspring were subjected to whole-genome resequencing analysis. A 940 kb deletion was identified at position 12621534–13561537 of chromosome V, with a 4 bp microhomology sequence (5′-TGCA-3′) present at the rejoined site (Figure 5A). Possible translocation of the deleted region to another location in the Ar50-33-pg1 genome was assessed using genomic PCR analysis of seven selected loci (Figure 5A,B). Expected amplicons were obtained with wild type template DNA, and no products were observed for the mutant, confirming deletion rather than translocation. PCR analysis also confirmed that removal of the 940 kb region in Ar50-33-pg1 resulted in rejoining of 5′ and 3′ flanking regions, corroborating the genome sequencing data.

A list of deleted genes in Ar50-33-pg1 is shown in Table S1. In total, 117 genes with AGI (Arabidopsis Genome Initiative) codes were absent, of which 41 were identified as protein-coding genes (Table 2). Several of these genes were previously characterized, including *AtPAP26* (At5g34850), *DD17* (At5g34885), *AtOS9* (At5g35080), and *EDR2* (At5g35180). Functionally uncharacterized genes included *AtMBD2* (At5g35330) and *AtMBD12* (At5g35338). Five genes encoded known chloroplast-targeted proteins, namely *TYRAAt1*/*TyrA1* (At5g34930), encoding arogenate dehydrogenase [53]; *AtCYP28* (At5g35100), encoding thylakoid lumen-localized cyclophilin [54]; *AMK5* (At5g35170), encoding an envelope- and thylakoid-associated adenylate kinase protein [55]; *PTM* (At5g35210), encoding an envelope-associated homeodomain protein [56]; and *EGY1* (At5g35220), encoding a thylakoid membrane-bound metalloprotease [17]. Loss-of-function phenotypes for these five genes were further investigated, on the basis that the impaired chloroplast structures in Ar50-33-pg1 might be derived from the loss of one or more of these proteins. The phenotype of *egy1*, which produced yellow green leaves and cotyledons in standard growth conditions [17,21,22], was similar to the phenotype of Ar50-33-pg1.

### 2.4. EGY1 Is the Main Causal Gene for the Pale Ar50-33-pg1 Phenotype

**Figure 6:**
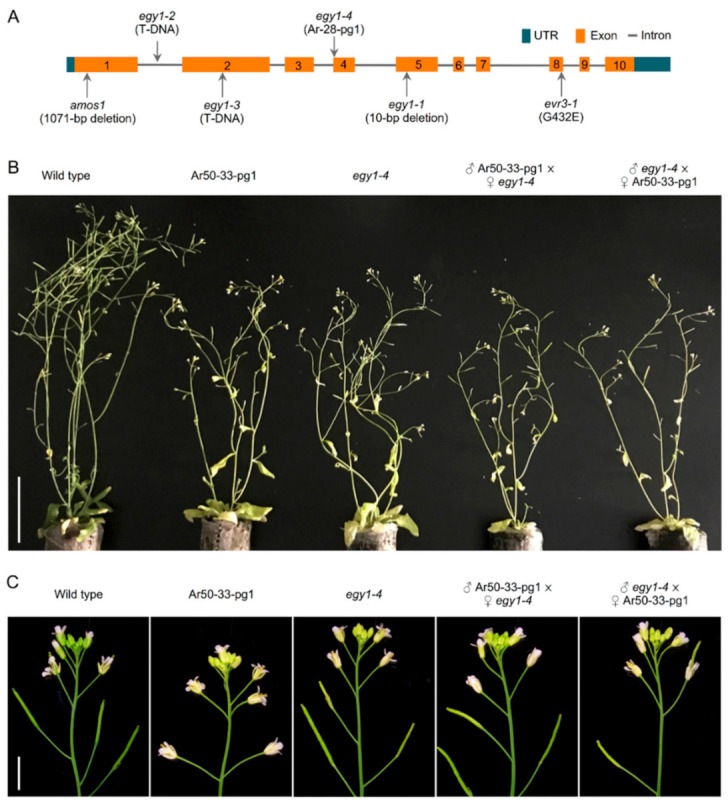
Characterization of F_1_ phenotypes of Ar50-33-pg1 and *egy1-4*. (**A**) Genomic structure of *EGY1*. Exons, introns, and six mutant alleles are indicated. One-month-old plants displaying (**B**) shoot morphology and (**C**) inflorescence. Bar = 8 cm (**B**) and 5 mm (**C**).

To test whether deletion of *egy1* was the primary cause of the Ar50-33-pg1 pale phenotype, the *egy1* mutant and Ar50-33-pg1 phenotypes were directly compared. An argon ion-derived line, Ar-28-pg1, was fortuitously obtained in our previous study [27] and used as the *egy1* mutant. The Ar-28-pg1 mutant carried small mutations (5 bp base substitutions and a 5 bp deletion) within 50 bp of the intron 3-exon 4 junction region of *EGY1* (Figure 6A). This allele was renamed *egy1-4* and, prior to its use, the following characteristics were confirmed: (i) Splicing defects were confirmed at the transcript level, predicting the production of C-terminally truncated proteins; (ii) No additional mutations were carried within the protein-coding region of *egy1-4*; (iii) F_1_ progeny of *egy1-4* and Col exhibited a normal green phenotype and F_2_ progeny exhibited the expected 3:1 ratio of normal green-to-pale green phenotypes. In addition, a similar phenotype to that of *egy1-4* was observed for a T-DNA insertion mutant, *egy1-2* [17]. Therefore, *egy1-4* was considered to be a loss-of-function mutant of *egy1* (Sanjaya et al., unpublished).

**Figure 7:**
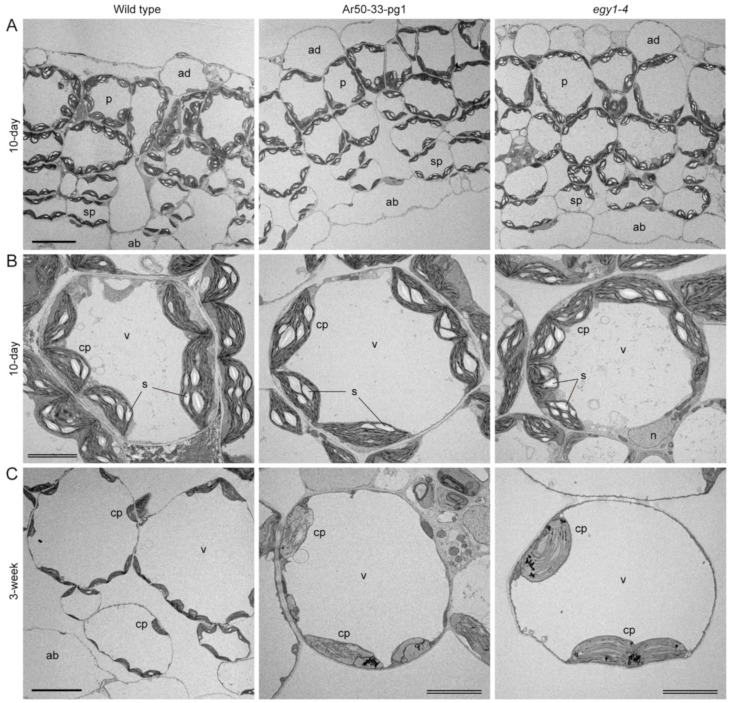
Ultrastructural analysis of leaf tissues and cells in wild type and mutant plants. Primary leaves of 10-day- and 3-week-old wild type, Ar50-33-pg1 and *egy1-4* plants analyzed by transmission electron microscopy. (**A**) Leaf tissues of 10-day-old plants. (**B**) Mesophyll cells of 10-day-old plants. (**C**) Mesophyll cells of 3-week-old plants. Abbreviations: ab, abaxial epidermis; ad, adaxial epidermis; cp, chloroplast; p, palisade cell; s, starch grain; sp, spongy cell; v, vacuole. Bar = 20 µm (single) and 5 µm (double).

The *egy1-4* mutant was characterized using the same methods as for Ar50-33-pg1, as shown in Figure 1, Figure 2 and Figure 3 and Figures S1–S4. The *egy1-4* mutant exhibited strikingly similar characteristics to Ar50-33-pg1 in terms of the moderate growth delay, reduced leaf-size, initial impairment of greening, and age-related chlorosis in photosynthetic tissues. The only differences observed between *egy1-4* and Ar50-33-pg1 were minor, with the growth delay and small leaf-size displaying slight recovery in *egy1-4*. Days to first flowering in *egy1-4* were 26.4 ± 1.2, compared with 29.8 ± 2.0 for Ar50-33-pg1 and 23.5 ± 1.2 for wild type (*n* = 10, for all). Importantly, many of the observed shoot morphologies were consistent with those described in earlier research [17]. Cellular chloroplast phenotypes were also consistent between *egy1-4* and Ar50-33-pg1 (Figure 4 and Figures S5–S7), with chloroplast lengths of 4–10 µm in 2-week-old samples (average, 6.4 µm; *n* = 30) and 3–6 µm in 4-week-old samples (average, 4.9 µm; *n* = 30). The major difference between *egy1-4* and Ar50-33-pg1 was that the abnormal flowering phenotypes observed in Ar50-33-pg1 (flower longevity, short siliques, and reduced seed production), were not observed in *egy1-4* (Figure S3 and Figure 6B,C). Finally, Ar50-33-pg1 was crossed with *egy1-4*, and the F_1_ progeny exhibited the same phenotypes as the parent lines (Figure 7B), with the exception that F_1_ progeny lost the abnormal flowering phenotypes of Ar50-33-pg1. This indicated that the reproductive defects in Ar50-33-pg1 were attributable to mutations other than *egy1*. Taken together, these data confirmed that loss of *EGY1* was the likely genetic cause of the pale phenotype in Ar50-33-pg1.

### 2.5. Mesophyll Chloroplasts of Ar50-33-pg1 and egy1-4 Exhibit Rapid Disassembly of Grana

Mesophyll chloroplasts of wild type, Ar50-33-pg1, and *egy1-4* were examined using transmission electron microscopy analysis. Primary leaves of 10-day- and 3-week-old seedlings were selected for analysis due to their synchronous emergence and growth among the three lines (Figure S1). Ten-day-old leaves corresponded to the chloroplast building stage, whereas 3-week-old leaves aligned with ongoing chlorosis and enabled observations of its various stages (Figure 3 and Figure S4).

At the tissue level, the differentiation of epidermis, palisade, and spongy tissues in all wild type, Ar50-33-pg1 and *egy1-4* leaves appeared normal (Figure 7A). Additionally, leaf mesophyll cells in all three 10-day-old samples had common typical features, including the large central vacuole and uniform lens-shaped chloroplasts attached to the surface of cell membranes (Figure 7B). Chloroplasts at this stage typically contained 2–6 starch grains per section. However, remarkable differences in mesophyll chloroplast structure were detected in 3-week-old leaves between the wild type and two mutants (Figure 7C). Importantly, consistent with the fluorescence microscopy results (Figure 4), the chloroplast interiors, but not the cellular configurations, differed dramatically.

**Figure 8:**
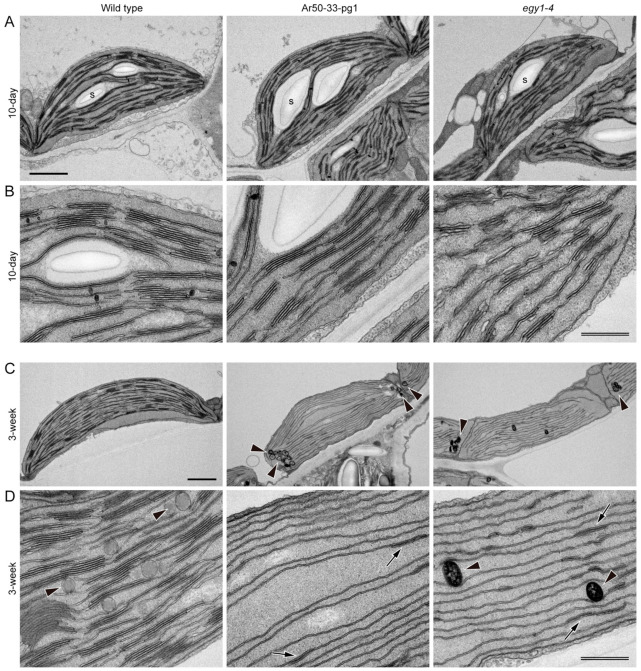
Ultrastructural analysis of mesophyll chloroplasts in wild type and mutant plants. Mesophyll cells of 10-day- and 3-week-old wild type, Ar50-33-pg1, and *egy1-4* plants analyzed by transmission electron microscopy. (**A**,**B**) Ten-day-old plants. (**C**,**D**) Three-week-old plants. Arrowheads and arrows indicate plastoglobules and short attachment sites between thylakoids, respectively. Abbreviation: s, starch grain. Bar = 1 µm (single) and 500 nm (double).

A closer look at the detailed chloroplast structure showed that the basic chloroplast structure of thylakoids, the stroma lamellae and the grana, which represent the non-appressed and appressed regions of thylakoids, respectively, was indistinguishable between wild type and the two mutants in 10-day-old leaves (Figure 8A,B). Although many of the chloroplasts were not fully developed at this stage, Ar50-33-pg1 and *egy1-4* chloroplasts both contained a well-developed thylakoid system. In 3-week-old leaves, wild type mesophyll chloroplasts exhibited developed grana, expanded size, and initial accumulation of spherical plastoglobules (Figure 8C). Mass production of plastoglobules is one of the structural features exhibited by degenerating chloroplasts upon leaf senescence [6,7]. Mutant chloroplasts from 3-week-old leaves displayed several novel features. First, grana had disappeared and only a single thylakoid layer was maintained (Figure 8C,D). These stroma lamellae-based thylakoids had an unusually long structure that extended in parallel between the chloroplast poles. The thylakoids aligned with chloroplast polarity, as in wild type, and the two single-layered thylakoids could form a short attachment site (Figure 8D, arrows). Only a small number of sparse interconnections were seen between these thylakoids. The single paralleled thylakoids occupied most of the chloroplast volume, which, alongside the almost complete absence of grana, indicated specific disassembly of the grana in thylakoids at an early stage of chloroplast degeneration. Chloroplasts of Ar50-33-pg1 and *egy1-4* both accumulated abundant plastoglobules with increased electron-dense staining compared with wild type chloroplasts (Figure 7C and Figure 8C, arrowheads). The plastoglobules in the two mutant chloroplasts tended to localize at the two polar chloroplast regions. Starch accumulation in the three plant lines was unclear in this analysis.

**Figure 9:**
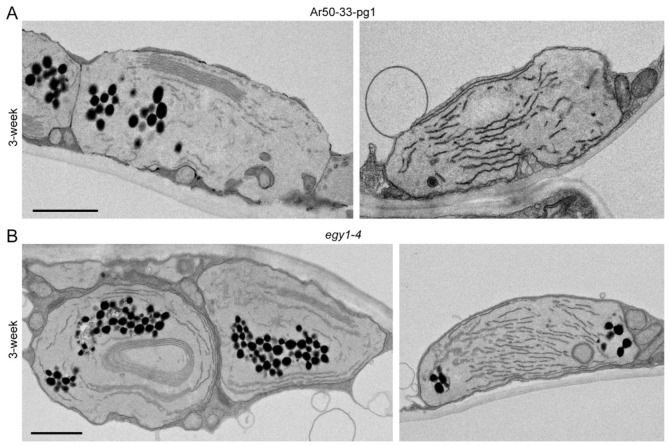
Ultrastructural analysis of mesophyll chloroplasts in mutant plants. Mesophyll chloroplasts of 3-week-old Ar50-33-pg1 and *egy1-4* plants with severe thylakoid degradation phenotypes analyzed by transmission electron microscopy. (**A**) Ar50-33-pg1. (**B**) *egy1-4*. Bar = 1 µm.

Additional advanced forms of thylakoid degradation were found in 3-week-old Ar50-33-pg1 and *egy1-4* mesophyll chloroplasts (Figure 9). One characteristic feature was the greater extent of thylakoid reduction, degradation, and fragmentation within shrunken chloroplasts (Figure 9), which resulted in a larger stromal space in the chloroplasts. Mass production of plastoglobules occurred in association with thylakoid degeneration, and these plastoglobules tended to form a cluster in the stroma. Furthermore, crepe-like, or even baumkuchen-like, multiple-layered thylakoids were frequently observed in the central or peripheral regions of the chloroplasts (Figure 9A,B). The mechanism underlying the formation of these seemingly ‘stacked’ thylakoids, which were not physically appressed but had an internal space, is unknown.

The structural abnormalities in thylakoids and plastoglobules were observed in both the Ar50-33-pg1 and *egy1-4* mutants (Figure 9). The reduced and occasionally fragmented thylakoids, absence of grana, and production of abundant plastoglobules were consistent with previous characterizations of *egy1-1*, *egy1-2*, and *egy1-3* chloroplasts [17].

Taken together, these observations revealed the importance of *EGY1* in the structural maintenance of grana, rather than in grana construction. Furthermore, the structural observations indicated the presence of an unknown mechanism for thylakoid organization after the disappearance of grana.

### 2.6. Leaf Chlorosis Is Accelerated by Nitrogen Starvation in Ar50-33-pg1 and egy1-4

**Figure 10:**
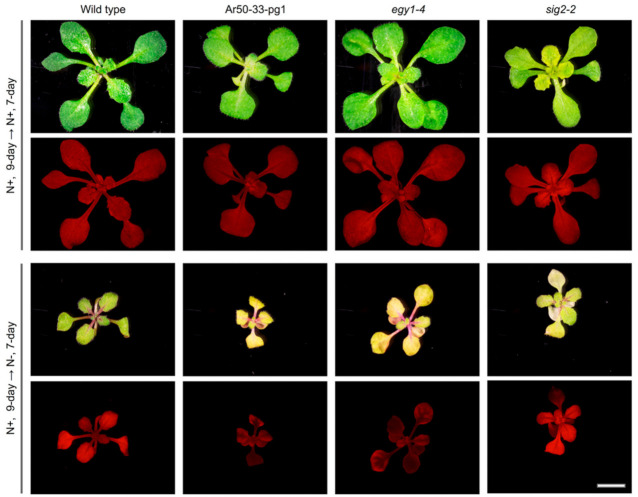
Plant growth analysis with nitrogen starvation. Bright-field and chlorophyll autofluorescence images of wild type, Ar50-33-pg1, *egy1-4*, and *sig2-2* plants are shown. Seedlings were cultured on MS agar medium for the first 9 days and then transferred to either normal or nitrogen-depleted MS medium for subsequent culture for 7 days. Bar = 5 mm.

The Ar50-33-pg1 and *egy1-4* mutants were assessed under nitrogen-starvation conditions. The *EGY1* gene is alternatively termed *AMOS1*. *AMOS1* was identified by a mutant screen for plants sensitive to high levels of NH_4_^+^ in the growth medium [19]. In addition, leaf chlorosis is known to occur under a range of physiological circumstances, including nutrient deficiency, prolonged dark exposure, and high-light irradiation. Nitrogen deprivation experiments are generally used to investigate the genes and processes of translocation, including nitrogen remobilization from sources to sinks [42,66,67], and we reasoned that Ar50-33-pg1 and *egy1-4* leaves might be responsive to nitrogen starvation. Wild type, Ar50-33-pg1, and *egy1-4* seedlings were cultivated on normal Murashige–Skoog (MS) media plates for 7 days and then transferred to nitrogen-deprived plates. Seedlings of another pale green mutant, *sig2-2*, were included as a control. Ar50-33-pg1 and *egy1-4* produced severely chlorotic leaves with lower chlorophyll fluorescence levels than wild type (Figure 10). The accelerated chlorosis was not evident in *sig2-2* seedlings, which nevertheless exhibited its inherent pale phenotype. Therefore, loss-of-function of *egy1* led to accelerated leaf chlorosis under nitrogen-deficient conditions as well as upon exposure to excess NH_4_^+^ [19].

## 3. Discussion

### 3.1. Heavy-Ion Beam-Induced Ar50-33-pg1 as a Resource for Plant Gene Analysis

In this study, a forward genetic screen was used to explore novel gene functions with importance for chloroplast activities. Heavy-ion beam mutagenesis, which induces mutations in a different way to EMS and T-DNA insertion technologies [26], was used to induce genomic mutations in *A. thaliana*. The Ar50-33-pg1 mutation, which exhibited a pale green foliar phenotype, was isolated and found to contain a 940 kb deletion on chromosome V. To our knowledge, along with several mutations induced by γ-ray-, carbon-ion-, and argon-ion-irradiations [30,68], this is one of the largest deletions maintained in a homozygous state in *A. thaliana*.

The Ar50-33-pg1 deleted region contained 117 missing genes, 41 of which were protein-coding genes. Approximately ten of these genes have been functionally characterized to date (Table 2 and Table S1). No stable genetic mutants are currently available for several of the characterized genes, and the Ar50-33-pg1 mutant might serve as a useful resource for analysis of these genes. Several possibilities were noted in our initial survey. These include *AtMBD12*, which encodes a methyl-CpG-binding domain protein 12, for which no T-DNA mutant is currently available. *AtMBD12* has a close paralog, *AtMBD2* [64,65], within a neighboring chromosomal region, and Ar50-33-pg1 is an *atmbd12 atmbd2* double knockout mutant. The physiological functions of *AtMBD12* and *AtMBD2* have not yet been described [64,65]. Another candidate is *TYRAAt1*/*TyrA1*, which encodes a chloroplast-targeted arogenate dehydrogenase [53,60]. Although gene-knockout lines for this gene are not yet available, knock-down lines induced impairments in anther dehiscence and seed production [60]. Although Ar50-33-pg1 was initially defective in seed production, the phenotype was recovered with plant aging, for unknown reasons (Figure S3; data not shown). The overlapping phenotype between *TyrA1* knock-down lines and Ar50-33-pg1 suggests that Ar50-33-pg1 could act as a reference for assessing *TYRAAt1*/*TyrA1* reproductive functions.

Five annotated genes that encoded chloroplast-targeted proteins were identified in the deleted region: *TYRAAt1*/*TyrA1* [53,60], *AtCYP28* [54,69], *AMK5* [55], *PTM* [56], and *EGY1* [17]. With the exception of *egy1*, inactivation of these genes reportedly produced chloroplast phenotypes that were indistinguishable from wild type. F_1_ progeny from a cross between Ar50-33-pg1 and *egy1-4* had a similar chloroplast phenotype to the parental lines. The major chloroplast phenotypes of Ar50-33-pg1 corresponded to those of *egy1-4*. This observation, alongside the apparent absence of chloroplast effects from individual inactivation of the other four chloroplast-related genes, indicates that the quadruple chloroplast gene knockout of Ar50-33-pg1 does not seriously impact chloroplast activities, at least under the laboratory growth conditions used in this study. Nevertheless, the slightly slower growth of Ar50-33-pg1 compared with *egy1-4* might be attributable to other missing Ar50-33-pg1 genes.

Ar50-33-pg1 had several abnormalities at the flowering stage that were not associated with *egy1* (Figure S3 and Figure 6). One or more of the absent genes from the deleted region may have been responsible for the reproductive phenotype. Several reproduction-related candidate genes were found in the deleted region of Ar50-33-pg1, including the female gametophyte-specific *DD17* and *ECA1* (gametogenesis gene)-related protein-coding genes, and the pollen-expressed *PICALM3* and *AtMBD2* genes.

### 3.2. Role of EGY1 Homologs in Plants

*EGY1* was previously identified by its mutant phenotype (*ethylene-dependent gravitropism-defective and yellow green*) in hypocotyls [17]. In the original characterization of *egy1* mutants, the yellow green phenotype of hypocotyls (cotyledons), seedlings, and leaves suggested that the gene was involved in chloroplast development. This hypothesis was supported by biochemical, cytological, and genetic analyses [17,22]. Transmission electron microscopy showed that fewer stroma lamellae and no grana were produced in mesophyll chloroplasts of 4-week-old *egy1* leaves. However, the cellular processes underlying the thylakoid deficiencies in leaves were unknown. In this study, *EGY1* was shown to have an additional important function in the maintenance of grana after chloroplast differentiation in leaf mesophyll cells.

Studies of *EGY1* homologs in other plant species may provide valuable insights into *EGY1* functions. *EGY1* belongs to the S2P protease gene family, which is widely found across prokaryotic and eukaryotic taxa. *EGY1* orthologs were found in tomato (*L2*) [70], tobacco (*WS1A* and *WS1B*) [71], and *Setaria* (*SiYGL2*) [72]. All the orthologs were necessary for maintenance of normal chlorophyll levels in a broad range of photosynthetic tissues, indicating a conserved function for plant *EGY1* orthologs. *A. thaliana* also has two distant paralogs of *EGY1*: *EGY2* and *EGY3*. All three *Arabidopsis* EGY proteins localize to the thylakoid membranes, and EGY1 and EGY2 (but not EGY3) possess ATP-independent proteolytic activities [17,73,74,75,76]. *EGY2* was found to be involved in hypocotyl elongation [75], and *EGY2* transcript levels were higher than those of *EGY1* in several developmental and physiological conditions [77]. However, the functional redundancy or divergency of the three *EGY* paralogs remains unclear and may be a future subject of analysis.

*A. thaliana EGY1* is the best characterized of the known plant *EGY* family members. Accumulated evidence indicates that this gene also has crucial functions for plant growth at the physiological level. A null *EGY1* mutant, *amos1* (*ammonium overly sensitive1*), exhibited accelerated chlorosis upon exposure to excess NH_4_^+^ [19] and also showed an increased resistance to phosphate deficiency [20]. In the current study, Ar50-33-pg1 and *egy1-4* plants exhibited accelerated chlorosis upon nitrogen deprivation (Figure 10). *EGY1* transcripts were abundantly detected in growing shoot tissues [18,76], and these mutant nutritional phenotypes might thus represent a secondary effect of the *egy1* mutation. Nevertheless, *EGY1* may be involved in the modulation of nitrogen and phosphate metabolism under conditions of plant nutritional stress. *EGY1* is also known as *evr3* (*enhancer of variegation3*) [22] due to its enhancement of the effects of mutant alleles of *VAR2*, which encodes a thylakoid membrane-bound metalloprotease FtsH2 [78,79]. Detailed biochemical analysis suggested that cooperation of the two thylakoidal metalloproteases might be needed for optimal photosynthesis [22]. These observations, alongside an apparent reduction in *EGY1* mRNA in older leaves, suggest that *EGY1* is a positive factor for both biogenesis and maintenance of chloroplasts, and that the *EGY1* mutations in Ar50-33-pg1 and *egy1-4* plants triggered the onset of the chloroplast dismantling processes prior to leaf maturation.

### 3.3. Cell Structural Mechanism of Leaf Chlorosis Caused by Loss of EGY1

One of the most important findings of this study was that the pale leaf phenotype in Ar50-33-pg1 (and *egy1-4*) involved disassembly of the grana in mesophyll chloroplasts. Ultrastructural analyses of the chloroplast destruction processes during *A. thaliana* leaf aging have been performed using various systems [5,80,81,82], and can be summarized as follows: (i) chlorophyll degradation; (ii) onset of volume reduction in chloroplasts and thylakoids together with the production of plastoglobules; (iii) continued chloroplast and thylakoid volume reductions accompanied by chloroplast rounding, preferential destruction of stroma lamellae, granal thickening, and plastoglobule accumulation; and (iv) chloroplast number reduction and maximal growth or accumulation of plastoglobules at the expense of thylakoids, leading to the formation of gerontoplasts [7]. In this study, Ar50-33-pg1 and *egy1-4* mesophyll chloroplasts followed similar chloroplast degeneration processes to those of leaf senescence, with the exception of the grana disassembly observed during chlorosis in the mutants (Figure 7, Figure 8 and Figure 9). However, there was no clear evidence of an association between rapid leaf chlorosis and leaf death (senescence) in mutants (Figure 2). Therefore, although stimulated by a different entry point, the chloroplast degeneration processes in *egy1*-deficient mesophyll cells were merged into a common pathway with the leaf senescence program.

The finding that *EGY1* plays a crucial role in grana structural maintenance provides insights for data interpretation (Figure 3 and Figure 4) and raises questions regarding the underlying cellular mechanisms. The balance between the activities of grana assembly (stacking) and disassembly (unstacking) in mesophyll chloroplasts may act as a structural determinant, and the identification of EGY1 proteolytic substrates in chloroplasts may provide further insight. Detailed cellular localization analysis of the EGY1 protein may prove informative, as previous chloroplast proteome analyses [74] suggest that cellular EGY1 levels may be very low. Several genes involved in thylakoid biogenesis have been identified by mutant studies, including factors essential for thylakoid formation (e.g., *ALB3* [83]) and crucial regulators of the thylakoid architecture (e.g., *FZL* [84,85], *GDC1* [86], *CURT1s* [87], and *RIQs* [88]). In addition, LHCII proteins and Photosystem II (e.g., LHCII trimer formation) are crucial for grana stacking (reviewed in [89]). EGY1 may degrade one or more of these proteins during normal metabolism to maintain the structural integrity of thylakoids and grana. Chemically modified proteins, including EGY1 itself [90], might also be the targets of proteolysis in this case.

From a cell morphological perspective, mutant and transgenic lines exhibiting either grana deficiency or overproduction may provide clues toward understanding *egy1*-induced grana disassembly. For example, *A. thaliana pcb2* [91] and *curt1abcd* [87] mutants formed thylakoids that were similarly devoid of appressed and clear-edged grana while retaining thylakoid stacking ability. This phenomenon led to the reticulated thylakoid pattern observed in two-dimensional ultrasections. Conversely, another *A. thaliana* mutant, *gdc1* [86], had defects in grana stacking that produced a ‘stroma thylakoids only’ appearance. The thylakoid structure of Ar50-33-pg1 and *egy1-4* in the chlorotic stage was similar to that of *gdc1*, in that long stretches of single thylakoids, which sometimes spanned chloroplasts from pole to pole, were arranged in parallel, and few thylakoid adhesions were observed (Figure 8). Only rudimentary grana stacks were detected in the Ar50-33-pg1 and *egy1-4* thylakoids (Figure 8). These examples raise questions regarding the relationships between the protein components or between the defective subcellular processes. Thylakoid regulation during the plant life cycle may provide further insight. Agranal thylakoids have been observed in leaf primordial plastids; cotyledon etioplasts have been observed just after light irradiation; and bundle sheath chloroplasts have been found specifically in C4 plants [2,3]. The *egy1* chloroplasts in chlorotic leaves strongly resembled the C4 bundle sheath chloroplasts, suggesting the intriguing possibility of a shared cellular mechanism.

## 4. Materials and Methods

### 4.1. Plant Materials and Growth Condition

*A. thaliana* (L.) Heynh. accession Columbia (Col) was used as wild type. The *egy1-4* mutant (originally designated Ar-28-pg1; Col background) was obtained by argon-ion irradiation in a previous study [27]. The *sig2-2* mutant (SALK_006646C; Col background) was a T-DNA insertion line generated at the Salk Institute [92] and obtained from the RIKEN Biological Resource Center (psy01036; Tsukuba, Japan). Seeds were surface sterilized and sown on Jiffy-7 (AS Jiffy Products, Stange, Norway) or 0.7% (*w*/*v*) agar-containing MS medium [93] (Wako, Osaka, Japan) supplemented with Gamborg’s B5 vitamins and 3% (*w*/*v*) sucrose. After vernalization at 4 °C for at least 2 days, germinated seedlings were grown under white light illumination with a long-day condition (05:00–21:00 [16 h light/8 h dark]; approximately 100 µE m^−2^ s^−1^) at 23 °C. For the nitrogen-starvation experiment, MS medium plates with (N+) or without (N−) nitrogen supplementation were used. In the latter, KNO_3_ was replaced by KCl, while NH_4_NO_3_ was removed. Seedlings were first grown on N+ plates for 9 days and then transferred onto either N+ or N− plates for additional growth for 7 days.

### 4.2. Heavy-Ion Beam Irradiation and Mutant Screening

Dry seeds of Col were exposed to ^40^Ar^17+^ ion radiation (290 keV µm^−1^ at a dose of 50 Gy) at the RIBF facility (RIKEN, Wako, Japan), as described by Kazama et al. [31,94]. The irradiated seeds (M_1_ generation) were sown and grown to maturity so that M_2_ seeds were amplified. In the M_2_ generation, seedlings underwent visual screening as described by Hirano et al. [27]. M_3_ seeds produced from M_2_ pools were then further examined with respect to the inheritance and consistency of phenotypes observed in the parental M_2_ plants. Among the M_3_ lines, the Ar50-33-pg1 mutant was chosen owing to the formation of pale green leaves and chlorophyll deficiency in guard cells of mature leaves (Sanjaya et al., unpublished observation), as seen using epifluorescence microscopy. Ar50-33-pg1 was twice backcrossed with Col for mutant characterization.

### 4.3. Epifluorescence Microscopy

Leaf tissues or cells were mounted under glass coverslips and observed using an Olympus IX71 microscope (Tokyo, Japan) equipped with a Hamamatsu Photonics ORCA-flash2.8 camera (Hamamatsu, Japan), using 60× water (N.A. 1.20) and 60× oil (N.A. 1.42) objective lenses (Olympus). Fluorescence signals of chlorophyll and SYBR Green (Lonza, Basel, Switzerland) were detected with BA575IF (Olympus) and FF01-545/55 (Semrock, Rochester, NY, USA) filters, respectively. Bright-field images were obtained with differential interference contrast (DIC) optics. In Figure 4, Figures S5 and S7, images of chlorophyll fluorescence were obtained using the same excitation and detection conditions for both the wild type and mutant samples. Digital black-and-white images were processed using ImageJ (v1.48; National Institutes of Health, Bethesda, MD, USA) or Photoshop (Adobe Systems, San Jose, CA, USA) to obtain pseudo-colored and final merged images.

### 4.4. Fluorescence Stereomicroscopy

Plant tissues were observed using a Leica MZ10 F microscope (Heidelberg, Germany) equipped with an Olympus DP26 camera, as described previously [95]. In Figure 1, Figure 2 and Figure 3, Figure 10, Figures S1, S2, S3D and S4, bright-field images and chlorophyll autofluorescence images were obtained using the same irradiation and detection conditions in both the wild type and mutant samples.

### 4.5. Protoplast Isolation and Organellar Nucleoid Staining

Leaf mesophyll protoplasts were isolated as described by Wu et al. [96]. First, the abaxial epidermis of leaves was removed using tape. The exposed mesophyll tissue was then immersed in enzyme solution containing 1% (*w*/*v*) Cellulase ‘Onozuka’ RS (Yakult, Tokyo, Japan), 0.25% (*w*/*v*) Macerozyme R-10 (Yakult), 0.4 M mannitol, 10 mM CaCl_2_, 20 mM KCl, 0.1% (*w*/*v*) BSA, and 20 mM MES. SYBR Green I (Lonza) was added at a dilution of 1:1000. Leaf samples were then placed in a rotary shaker (40 rpm) for 60 min at 23 °C to allow digestion of mesophyll cell walls and nucleoid staining. The resulting protoplast suspension was mounted under glass coverslips and observed using epifluorescence microscopy as described above.

### 4.6. Measurement of Leaf Chlorophylls

Approximately 100 mg of fresh leaves were harvested from 4-week-old plants grown on Jiffy-7. Leaf samples were soaked in 3 mL *N*,*N*-dimethylformamide (Nacalai Tesque, Kyoto, Japan) overnight at −25 °C. After exclusion of plant debris, the supernatant was transferred to a glass cuvette, and absorbance was measured using a spectrophotometer (Shimadzu UV-1240; Kyoto, Japan) at 663.8 and 646.8 nm wavelengths. Levels of chlorophyll *a* and *b* were determined by the formula of Porra et al. [43]: Chl *a* (µg mL^−1^) = 12.00 × *A*_663.8_ − 3.11 × *A*_646.8_; Chl *b* (µg mL^−1^) = 20.78 × *A*_646.8_ − 4.88 × *A*_663.8_.

### 4.7. Whole-Genome Resequencing Analysis and Determination of Mutation Locus

Genomic DNA was extracted from leaves using a DNeasy Plant Mini Kit (QIAGEN, Hilden, Germany). The extracted DNA was sequenced by Macrogen Japan (Kyoto, Japan) using a HiSeq 4000 sequencing system (Illumina Inc., San Diego, CA, USA; https://www.illumina.com (accessed on 21 April 2021)) as described previously [30,31]. The obtained reads were processed using an automated mutation analysis pipeline (AMAP), as described previously [97], and the Arabidopsis TAIR10 release was used as the reference genome. The detected putative mutations, except those derived from mitochondria and chloroplasts, were further screened for false positives by comparing the detected mutations in several previously reported heavy-ion mutant lines [31]. Mutations that were shared across mutant lines were regarded as false positives and removed from the list of candidates. Next, the candidate mutations were examined manually using the Integrative Genomics Viewer (IGV) [98]. Finally, the single remaining mutation candidate (a 940 kb deletion) was confirmed through genomic PCR (primers are listed in Table S2).

### 4.8. Electron Microscopy

Primary leaves of 10-day- and 3-week-old wild type, Ar50-33-pg1, and *egy1-4* seedlings grown on Jiffy-7 were harvested from 10:30 to 12:00 am. After removing the midrib region using razors, samples were fixed with 2% paraformaldehyde and 2% glutaraldehyde in 0.05 M cacodylate buffer pH 7.4 at 4 °C overnight. After primary fixation, ultrastructural analysis of leaf cells was performed by Tokai Electron Microscopy Inc. (Nagoya, Japan). The samples were washed three times with 0.05 M cacodylate buffer for 30 min each, and were postfixed with 2% osmium tetroxide (OsO_4_) in 0.05 M cacodylate buffer at 4 °C for 3 h. The samples were dehydrated in graded ethanol solutions followed by infiltration with propylene oxide and resin (Quetol-651; Nisshin EM Co., Tokyo, Japan). The polymerized resins were ultra-thin sectioned at 80 nm with a diamond knife using an ultramicrotome (Ultracut, UCT; Leica, Vienna, Austria), and the sections were mounted on copper grids. Mounted sections were stained with 2% uranyl acetate at room temperature for 15 min, washed with distilled water, and then treated with secondary lead stain solution (Sigma-Aldrich Co., Tokyo, Japan) at room temperature for 3 min. Grids were observed using a transmission electron microscope (JEM-1400Plus; JEOL, Ltd., Tokyo, Japan) at an accelerated voltage of 100 kV. Digital images (3296 × 2472 pixels) were obtained using a CCD camera (EM-14830RUBY; JEOL, Ltd.).

## Figures and Tables

**Table 1:** Segregation analysis of Ar50-33-pg1. Phenotypes of F_1_ and F_2_ seedlings after growth on MS media for 14 days. The pale green phenotype at the seedling stage was also observed in the flowering stage.

Cross	Generation	Number of Plants			Expected Ratio (Normal: Pale Green)	Chi-Square Value
		Total	Normal	Pale Green		
♂Ar50-33-pg1 * × ♀Col	F_1_	51	51	0	1:0	0.00
♂Ar50-33-pg1 * × ♀Col	F_2_	185	145	40	3:1	1.13
♂Col × ♀Ar50-33-pg1 **	F_1_	40	40	0	1:0	0.00
♂Col × ♀Ar50-33-pg1 **	F_2_	477	374	103	3:1	2.95

* M_3_ generation used. ** First backcross generation used.

**Table 2:** List of protein-coding genes deleted in Ar50-33-pg1. AGI codes and gene descriptions were compiled from the TAIR site (https://www.arabidopsis.org, accessed on 21 April 2021). References are provided for characterized genes. The feature/localization column contains experimentally determined information or the localization or protein signature predicted by TargetP software (http://www.cbs.dtu.dk/services/TargetP/, accessed on 21 April 2021) (probability in bracket). Full details of deleted genes are in Table S1.

AGI Code	Feature/Localization	Description/Function	Reference
AT5G33390	Other (1.0)	glycine-rich protein	
AT5G33393	Other (1.0)	myosin heavy chain-like protein	
AT5G33406	Mitochondrion (0.9)	hAT dimerization domain-containing protein/transposase-like protein	
AT5G33806	Other (1.0)	hypothetical protein	
AT5G33898	Other (1.0)	hypothetical protein	
AT5G34581	Other (0.9)	hydroxyproline-rich glycoprotein family protein	
AT5G34780	Other (1.0)	putative ketopantoate reductase (KPR)	[57]
AT5G34828	Signal peptide (1.0)	a Plant thionin family protein	
AT5G34829	Other (0.8)	transmembrane protein	
AT5G34830	Other (1.0)	hypothetical protein	
AT5G34850	Lytic vacuole	AtPAP26; a root-secreted purple acid phosphatase precursor involved in extracellular phosphate-scavenging	[58]
AT5G34869	Other (0.8)	hypothetical protein	
AT5G34870	Other (1.0)	zinc knuckle (CCHC-type) family protein	
AT5G34882	Signal peptide (1.0)	a ECA1 gametogenesis related family protein	
AT5G34883	Signal peptide (1.0)	inhibitor/lipid-transfer protein/seed storage 2S albumin superfamily protein	
AT5G34885	Signal peptide (1.0)	DD17; inhibitor/lipid-transfer protein/seed storage 2S albumin superfamily protein	[59]
AT5G34887	Signal peptide (1.0)	inhibitor/lipid-transfer protein/seed storage 2S albumin superfamily protein	
AT5G34905	Signal peptide (1.0)	ECA1 gametogenesis family protein	
AT5G34908	Signal peptide (1.0)	a ECA1 gametogenesis related family protein	
AT5G34930	Chloroplast stroma	TYRAAt1/TyrA1; arogenate dehydrogenase	[53,60]
AT5G34940	Signal peptide (1.0)	ATGUS3/GUS3; glucuronidase 3	[61]
AT5G35067	Other (1.0)	hypothetical protein	
AT5G35069	Other (0.6), Mitochondrion (0.3)	a small protein and has either evidence of transcription or purifying selection	
AT5G35080	Endoplasmic reticulum	ATOS9/OS9; a protein involved in the endoplasmic reticulum-associated degradation of glycoproteins	[62]
AT5G35090	Other (1.0)	hypothetical protein	
AT5G35100	Chloroplast thylakoid lumen	AtCYP28; Cyclophilin-like peptidyl-prolyl cis-trans isomerase family protein	[54]
AT5G35110	Other (1.0)	hypothetical protein	
AT5G35120	Other (1.0)	MADS-box family protein	
AT5G35160	Signal peptide (1.0)	TMN11; Endomembrane protein 70 protein family	
AT5G35170	Chloroplast envelope and thylakoid membranes	AMK5; adenylate kinase family protein	[55]
AT5G35180	Mitochondrion	EDR2; ENHANCED DISEASE RESISTANCE protein	[63]
AT5G35190	Signal peptide (1.0)	EXT13; proline-rich extensin-like family protein	
AT5G35195	Other (0.9)	a defensin-like (DEFL) family protein	
AT5G35200	Other (1.0)	PICALM3; ENTH/ANTH/VHS superfamily protein	
AT5G35210	Chloroplast envelope membrane	PTM/DDP1; PHD type transcription factor with transmembrane domains/DDT-PHD protein1	[56]
AT5G35220	Chloroplast thylakoid membrane	EGY1/AMOS1/EVR3; ethylene-dependent gravitropism-deficient and yellow-green 1/ammonium overly sensitive 1/enchancer of variegation 3	[17,18,19,20,21,22]
AT5G35230	Other (1.0)	hypothetical protein	
AT5G35300	Other (0.9)	hypothetical protein	
AT5G35320	Other (1.0)	DBH-like monooxygenase	
AT5G35330	Other (1.0)	ATMBD2/MBD2; methyl-cpg-binding domain protein 02	[64,65]
AT5G35338	Other (1.0)	MBD12; methyl-cpg-binding domain protein 12	

## Data Availability

Nucleotide sequence data files are available in the DDBJ Sequenced Read Archive under the accession number DRA011751.

## Supplementary Materials

The following are available online at https://www.mdpi.com/article/10.3390/plants10050848/s1, Figure S1: Early seedling morphology of 10-day-old wild type and mutant plants, Figure S2: Stem phenotypes of wild type and mutant plants, Figure S3: Flowering phenotypes of wild type and mutant plants, Figure S4: Fluorescence stereomicroscopy of leaf chlorosis, Figure S5: Fluorescence microscopy of leaf mesophyll protoplasts from wild type and mutant plants stained with SYBR Green, Figure S6: Light microscopy of leaf mesophyll protoplasts from wild type and mutant plants, Figure S7: Fluorescence microscopy of unstained leaf mesophyll protoplasts from wild type and mutant plants, Table S1: Full list of deleted genes in Ar50-33-pg1, Table S2: List of primers used for genomic PCR analysis of Ar50-33-pg1.
