# Supplementary material for: An Argon-Ion-Induced Pale Green Mutant of Arabidopsis Exhibiting Rapid Disassembly of Mesophyll Chloroplast Grana
Source: Plants (Basel). 2021 Apr 22;10(5):848. doi: 10.3390/plants10050848 (PMC8145761; doi:10.3390/plants10050848)
Supplement: Supplementary file 1 [file plants-10-00848-s001.zip › plants-1187874-supplementary.pdf]

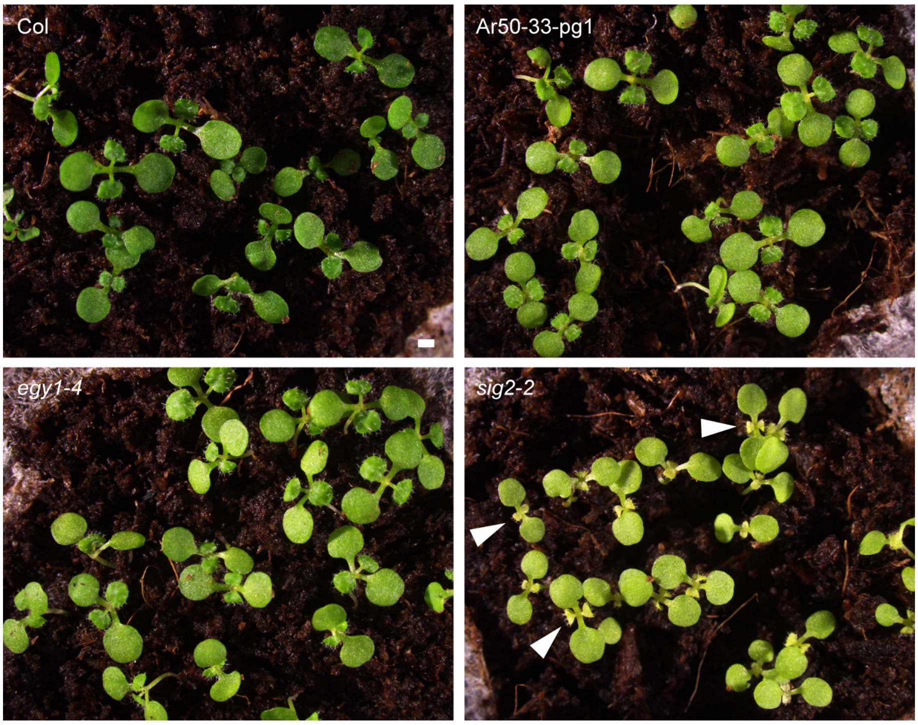

Figure S1. Early seedling morphology of 10-day-old wild type and mutant plants. Images of wild type, Ar50-33-pg1, *egy1-4*, and *sig2-2* seedlings are shown. Primary leaves of *sig2-2* exhibited delayed growth and greening (arrowheads). Bar = 1 mm.

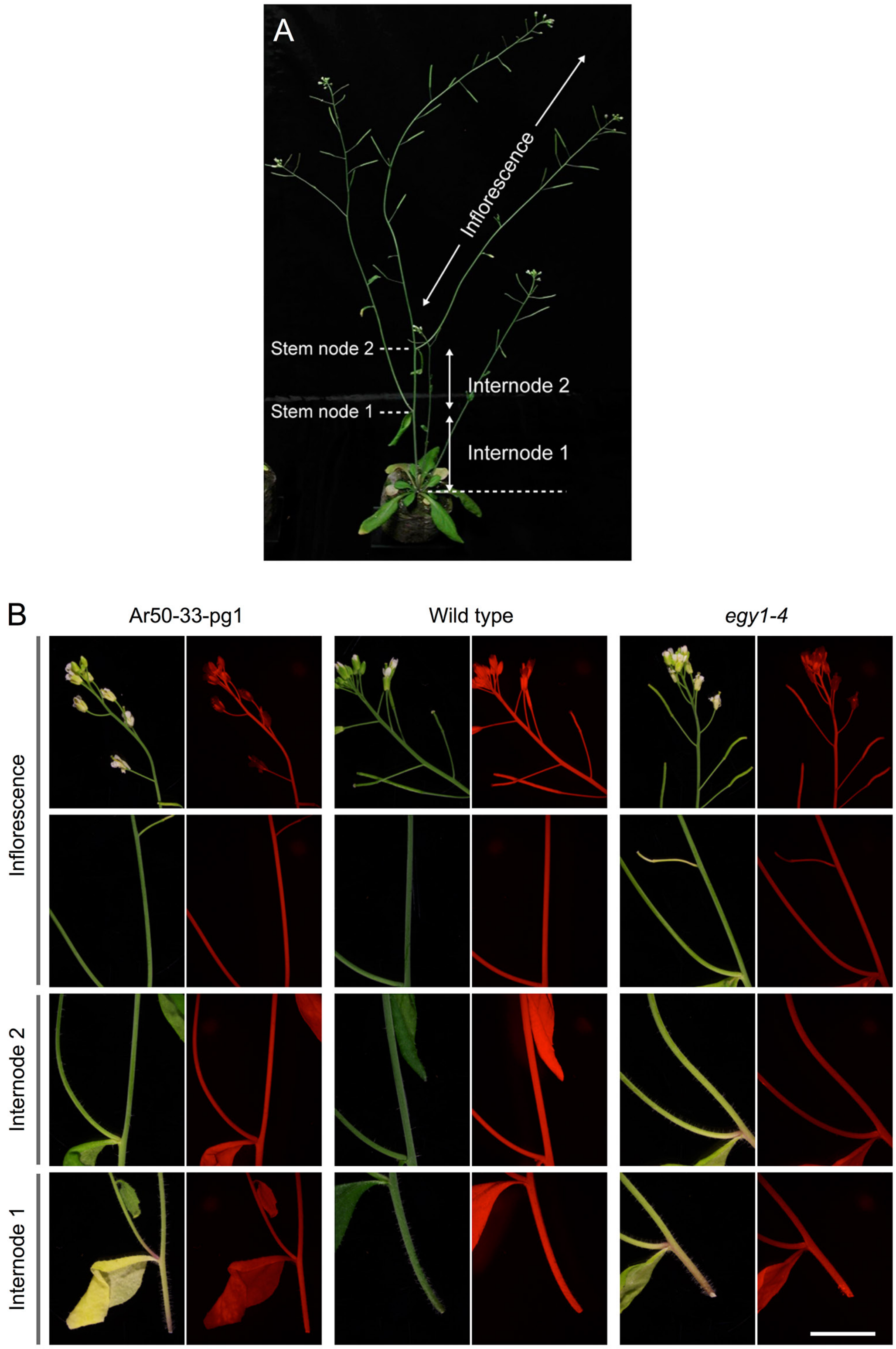

Figure S2. Stem phenotypes of wild type and mutant plants. (**A**) Stem terminology. (**B**) Images of bright-field (left panels) and chlorophyll autofluorescence (right panels) at internode 1, internode 2, and inflorescence from the main axis of Ar50-33-pg1 (4-week-old), wild type (5-week-old), and *egy1-4* (4-week-old) stems. Bar = 1 cm.

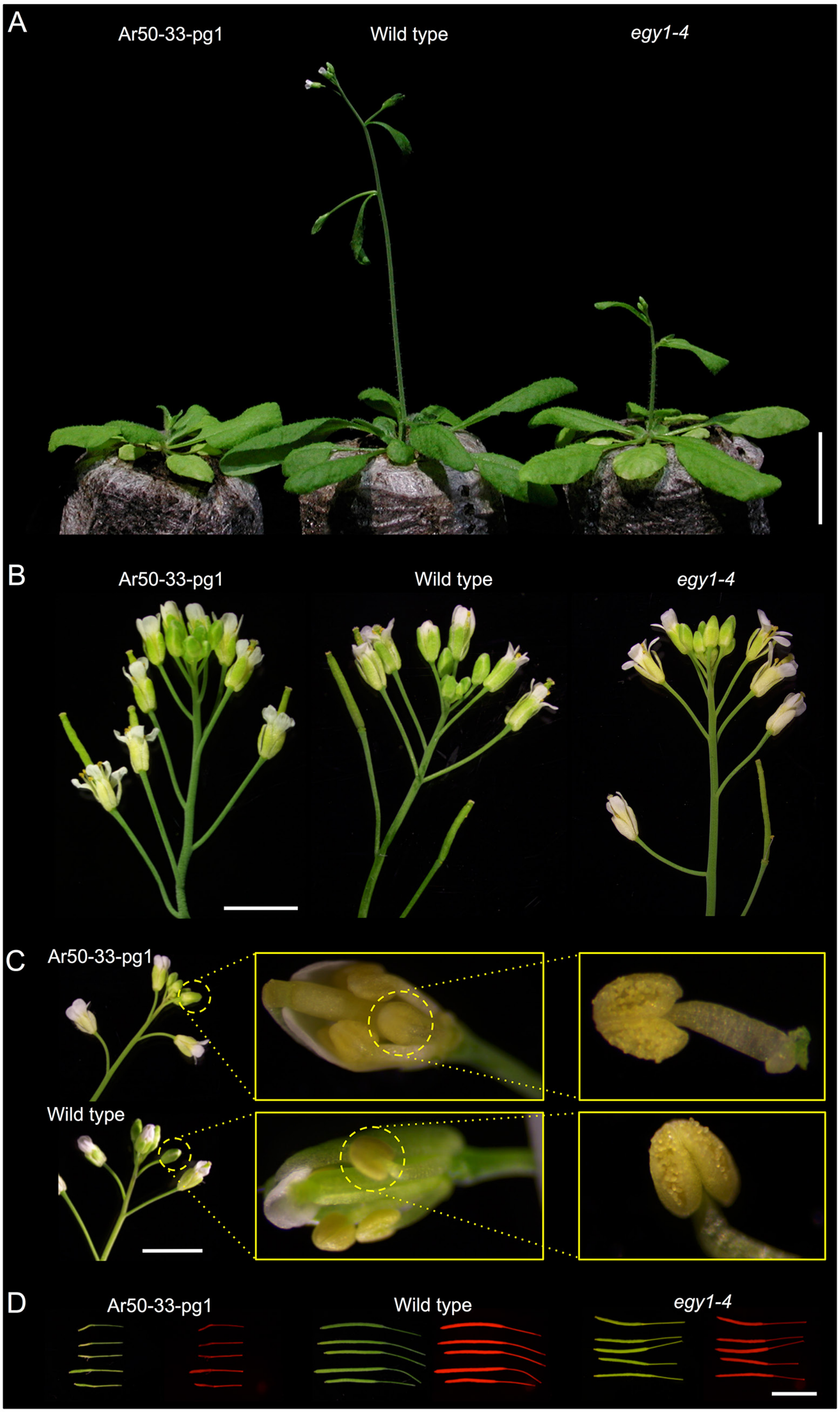

Figure S3. Flowering phenotypes of wild type and mutant plants. (**A**) 24-day-old Ar50-33-pg1, wild type, and *egy1-4* plants. (**B**) Inflorescence. (C) Developing flower and stamen. (**D**) Silique. In (**D**), bright-field and chlorophyll autofluorescence images were obtained using 5-week-old plants. Bar = 2 cm (**A**), 5 mm (**B**,**D**) and 1 cm (**C**).

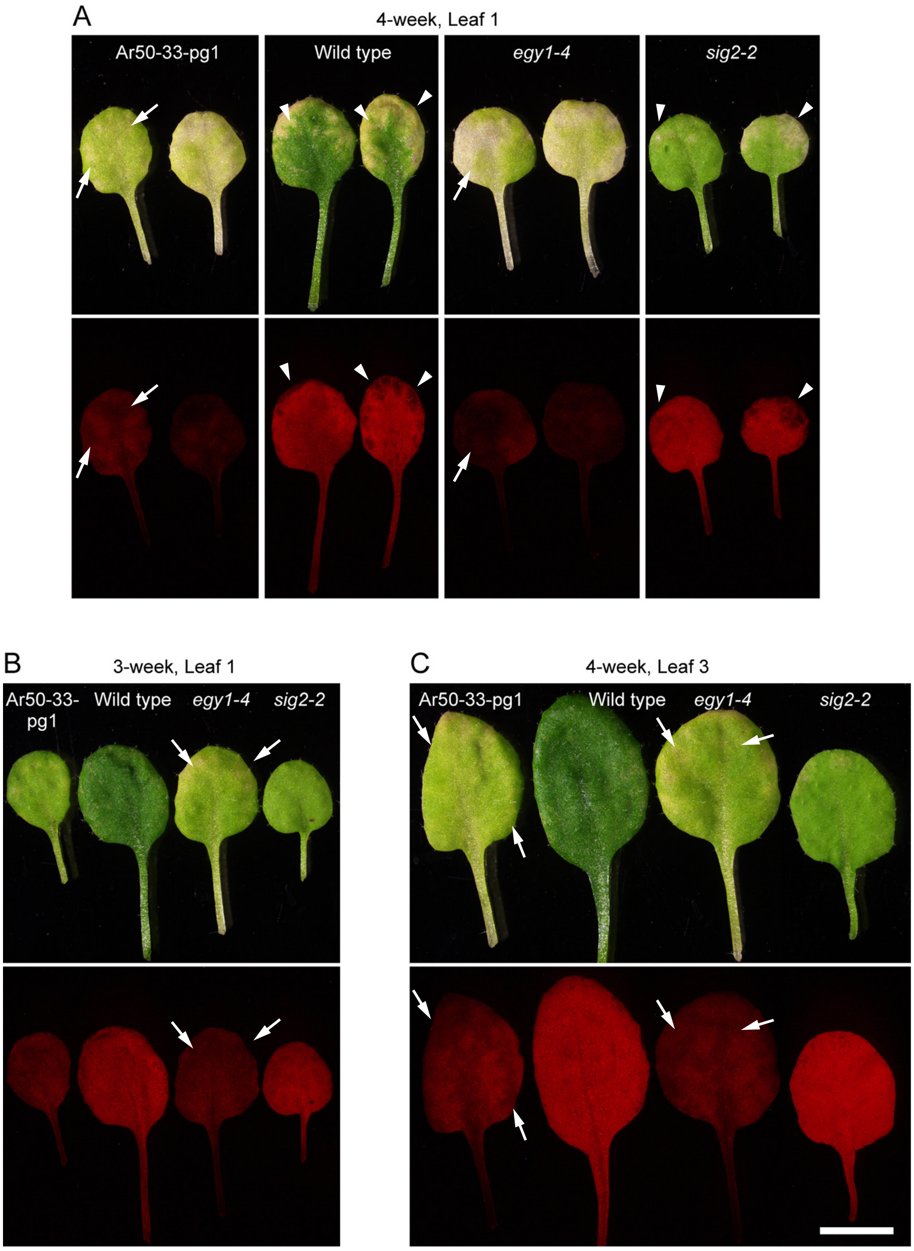

Figure S4. Fluorescence stereomicroscopy of leaf chlorosis. (**A**) Primary leaves from 4-week-old seedlings. (**B**) Primary leaves from 3-week-old seedlings. (**C**) The third leaves from 4-week-old seedlings. In (**A–C**), bright-field and chlorophyll autofluorescence images were taken using Ar50-33-pg1, wild type, *egy1-4*, and *sig2-2* plants. Arrowheads indicate the initiation of chlorosis and subsequent necrosis at the leaf tip and margin in wild type and *sig2-2.* Arrows indicate the occurrence of chlorosis in a patchy pattern across the leaf blade in Ar50-33-pg1 and *egy1-4*. Bar = 5 mm.

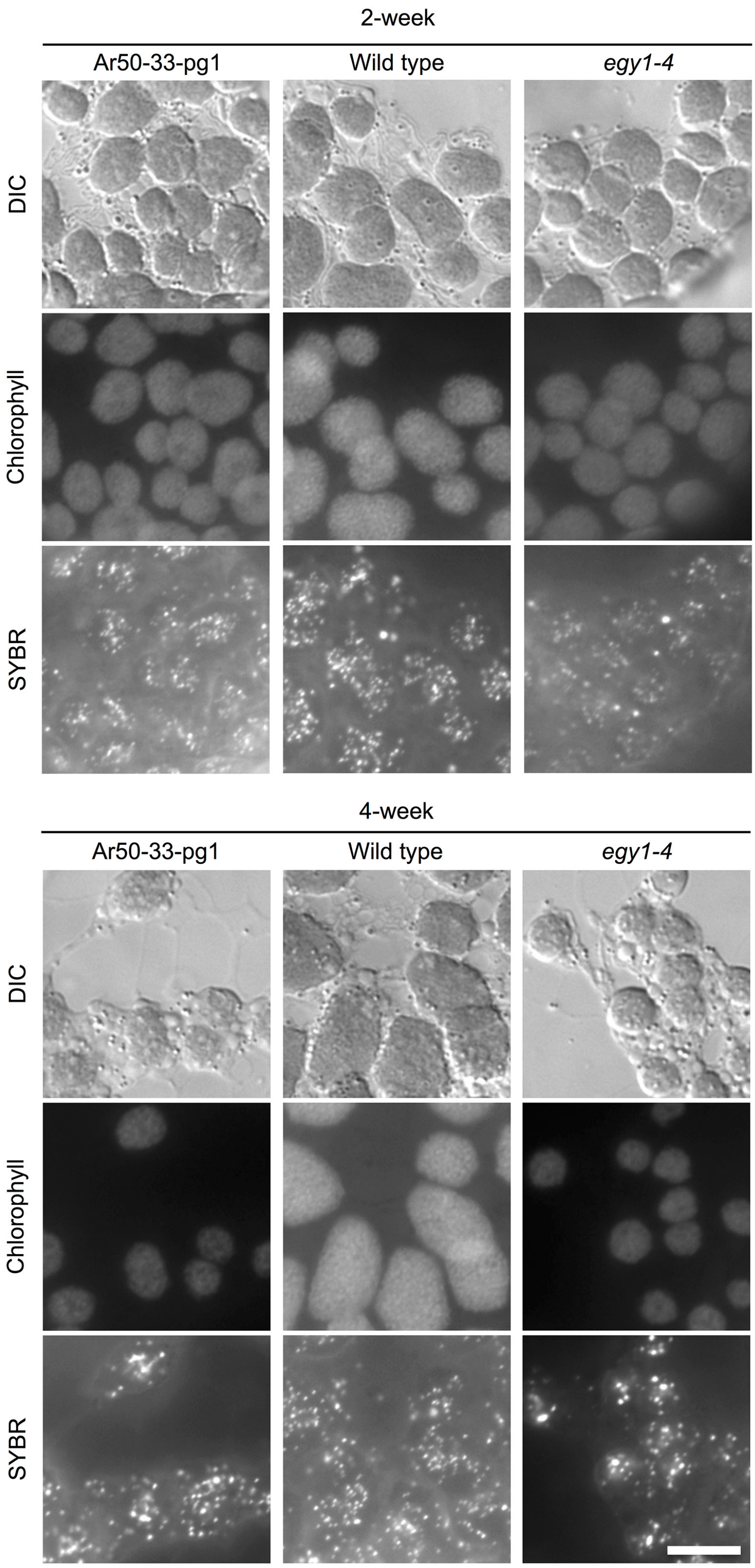

Figure S5. Fluorescence microscopy of leaf mesophyll protoplasts from wild type and mutant plants stained with SYBR Green. Protoplasts from primary leaves of 2- and 4-week-old wild type, Ar50-33-pg1 and *egy1-4* plants were stained with SYBR Green. Images of DIC, chlorophyll autofluorescence, and SYBR Green fluorescence are shown. See also Figure 4. Bar = 10 µm.

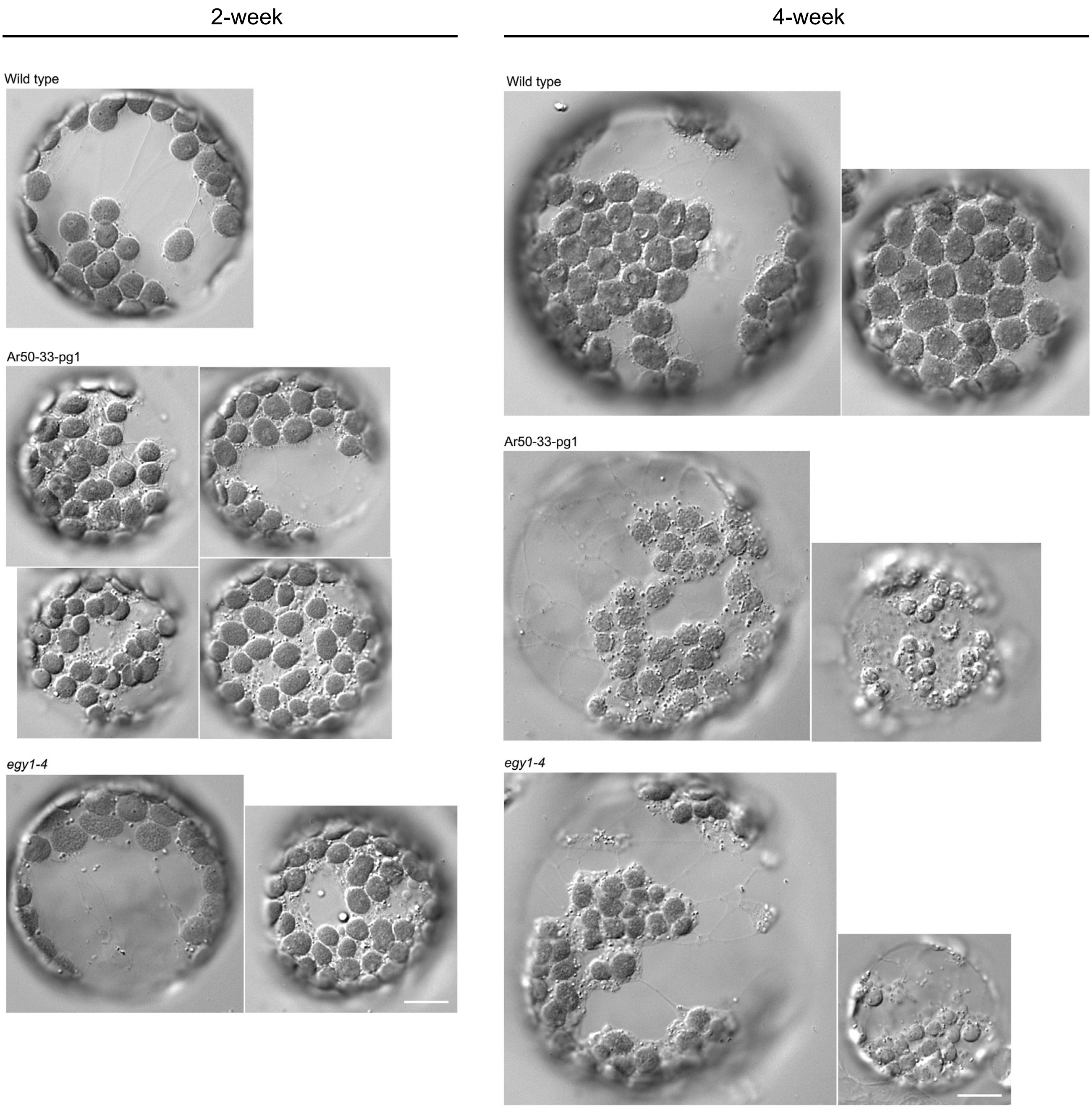

Figure S6. Light microscopy of leaf mesophyll protoplasts from wild type and mutant plants. Images of protoplasts from primary leaves of 2- and 4-week-old wild type, Ar50-33-pg1 and *egy1-4* plants were taken with DIC optics. See also Figure 4. Bar = 10 µm.

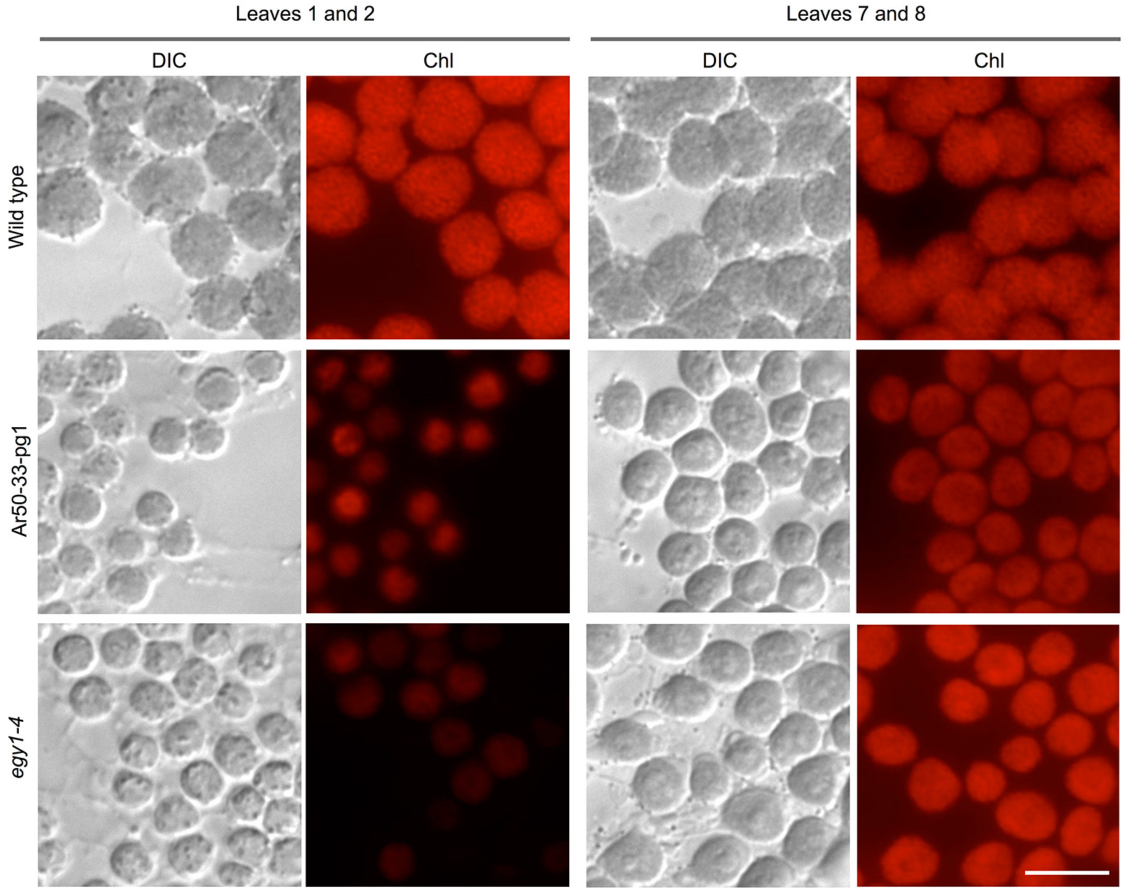

Figure S7. Fluorescence microscopy of unstained leaf mesophyll protoplasts from wild type and mutant plants. Protoplasts from young (leaves 7 and 8) and old leaves (leaves 1 and 2) from 4-week-old wild type, Ar50-33-pg1, and *egy1-4* plants. Images of DIC and chlorophyll autofluorescence are shown. See also Figure 4. Bar = 10 µm.

Supplementary Table S1. Full list of deleted genes in Ar50-33-pg1. AGI codes and descriptions of each gene were compiled from the TAIR website (https://www.arabidopsis.org). See also Table 2.

AGI code	Feature/ localization	Description/ function
AT5G33383.1		transposable_element_gene
AT5G33384.1		transposable_element_gene
AT5G33385.1		transposable_element_gene
AT5G33386.1		transposable_element_gene
AT5G33387.1		transposable_element_gene
AT5G33388.1		transposable_element_gene
AT5G33389.1		transposable_element_gene
AT5G33391.1		transposable_element_gene
AT5G33392.1		transposable_element_gene
AT5G33393.1	Other (1.0)	myosin heavy chain-like protein
AT5G33390.1	Other (1.0)	glycine-rich protein
AT5G33395.1		transposable_element_gene
AT5G33398.1		transposable_element_gene
AT5G33402.1		transposable_element_gene
AT5G33404.1		transposable_element_gene
AT5G33406.1	Mitochondrion (0.9)	hAT dimerization domain-containing protein / transposase-like protein
AT5G05075.1		Long non-coding RNA
AT5G05095.1		Long non-coding RNA
AT5G33399.1		Long non-coding RNA
AT5G33400.1		pseudogenic_transcript
AT5G33405.1		pseudogenic_transcript
AT5G33410.1		transposable_element_gene
AT5G33415.1		transposable_element_gene
AT5G33420.1		transposable_element_gene
AT5G33422.1		transposable_element_gene
AT5G33424.1		transposable_element_gene
AT5G33427.1		transposable_element_gene
AT5G33428.1		transposable_element_gene
AT5G33432.1		pseudogenic_transcript -> transposable_element_gene similar to En/Spm-like transposon
AT5G33436.1		pseudogene of Ribonuclease H-like superfamily protein
AT5G33441.1		pseudogenic_transcript
AT5G33439.1		long non-coding RNA
AT5G33433.1		transposable_element_gene
AT5G33431.1		pseudogene
AT5G33434.1		transposable_element_gene
AT5G33438.1		transposable_element_gene
AT5G33442.1		pseudogene
AT5G33533.1		transposable_element_gene
AT5G33624.1		transposable_element_gene
AT5G33715.1		transposable_element_gene
AT5G33806.1	Other (1.0)	hypothetical protein
AT5G33898.1	Other (1.0)	hypothetical protein
AT5G33990.1		transposable_element_gene
AT5G34082.1		transposable_element_gene
AT5G34174.1		transposable_element_gene
AT5G34358.1		transposable_element_gene
AT5G34266.1		transposable_element_gene
AT5G34376.1		transposable_element_gene
AT5G34394.1		transposable_element_gene
AT5G34412.1		transposable_element_gene
AT5G34431.1		transposable_element_gene
AT5G34450.1		transposable_element_gene
AT5G34480.1		transposable_element_gene
AT5G34500.1		transposable_element_gene
AT5G34520.1		transposable_element_gene
AT5G34540.1		transposable_element_gene
AT5G05115.1		novel transcribed region
AT5G05115.2		novel transcribed region
AT5G05115.3		novel transcribed region
AT5G34602.1		transposable_element_gene

AT5G34644.1		transposable_element_gene
AT5G34623.1		transposable_element_gene
AT5G34665.1		transposable_element_gene
AT5G34686.1		transposable_element_gene
AT5G34696.1		transposable_element_gene
AT5G34707.1		transposable_element_gene
AT5G34728.1		transposable_element_gene
AT5G34749.1		transposable_element_gene
AT5G34770.1		transposable_element_gene
AT5G34560.1		pseudogene of uridine kinase-like 4
AT5G34581.1	Other (0.9)	hydroxyproline-rich glycoprotein family protein
AT5G34780.1	Other (1.0)	putative ketopantoate reductase (KPR)
AT5G34825.1		transposable_element_gene
AT5G34790.1		transposable_element_gene
AT5G34795.1		pseudogene
AT5G34800.1		transposable_element_gene
AT5G34810.1		pseudogene
AT5G34820.1		transposable_element_gene
AT5G34830.1	Other (1.0)	hypothetical protein
AT5G34829.1	Other (0.8)	transmembrane protein
AT5G34831.1		pseudogene of myosin heavy chain-like protein
AT5G34832.1		pseudogene of hypothetical protein
AT5G34833.1		transposable_element_gene
AT5G34834.1		transposable_element_gene
AT5G34835.1		transposable_element_gene
AT5G34839.1		transposable_element_gene
AT5G34842.1		transposable_element_gene
AT5G34843.1		transposable_element_gene
AT5G34844.1		transposable_element_gene
AT5G34845.1		transposable_element_gene
AT5G34846.1		transposable_element_gene
AT5G34847.1		transposable_element_gene
AT5G34840.1		transposable_element_gene
AT5G34848.1		pseudogene
AT5G34849.1		transposable_element_gene
AT5G34869.1	Other (0.8)	hypothetical protein
AT5G34828.1	Signal peptide (1.0)	a Plant thionin family protein
AT5G05125.1		long non-coding RNA
AT5G34871.1		long non-coding RNA
AT5G34851.1		transposable_element_gene
AT5G34852.1		pseudogene
AT5G34853.1		transposable_element_gene
AT5G05135.1		long non-coding RNA
AT5G05145.4<br>AT5G05145.3<br>AT5G05145.2		novel transcribed region
AT5G34850.1	Lytic vacuole	AtPAP26; a root-secreted purple acid phosphatase precursor involved in extracellular phosphate-scavenging
AT5G34854.1		transposable_element_gene
AT5G34855.1		transposable_element_gene
AT5G34856.1		transposable_element_gene
AT5G34857.1		transposable_element_gene
AT5G34858.1		transposable_element_gene
AT5G34859.1		transposable_element_gene
AT5G34861.1		transposable_element_gene
AT5G34862.1		transposable_element_gene
AT5G34863.1		transposable_element_gene
AT5G34864.1		transposable_element_gene
AT5G34865.1		transposable_element_gene
AT5G34866.1		transposable_element_gene
AT5G34867.1		transposable_element_gene
AT5G34868.1		transposable_element_gene
AT5G34860.1		transposable_element_gene
AT5G34870.1	Other (1.0)	zinc knuckle (CCHC-type) family protein
AT5G34880.1		transposable_element_gene
AT5G34882.1	Signal peptide (1.0)	a ECA1 gametogenesis related family protein
AT5G34883.1	Signal peptide (1.0)	inhibitor/lipid-transfer protein/seed storage 2S albumin superfamily protein

AT5G34885.1	Signal peptide (1.0)	DD17; inhibitor/lipid-transfer protein/seed storage 2S albumin superfamily protein
AT5G34887.1	Signal peptide (1.0)	inhibitor/lipid-transfer protein/seed storage 2S albumin superfamily protein
AT5G34895.1		transposable_element_gene
AT5G34900.1		transposable_element_gene
AT5G34903.1		ECA1 gametogenesis related family protein [pseudogene] (TAIR)
AT5G34905.1	Signal peptide (1.0)	ECA1 gametogenesis family protein
AT5G34908.1	Signal peptide (1.0)	a ECA1 gametogenesis related family protein
AT5G34910.1		pseudogene of hypothetical protein
AT5G34920.1		transposable_element_gene
AT5G34925.1		transposable_element_gene
AT5G34930.1<br>AT5G34930.2<br>AT5G34930.3	Other (1.0)	TYRAAt1/TyrA1; arogenate dehydrogenase
AT5G34940.1<br>AT5G34940.2<br>AT5G34940.3<br>AT5G34940.4	Signal peptide (1.0)	ATGUS3/GUS3; glucuronidase 3
AT5g34945.1		pseudogene
AT5G34950.1		transposable_element_gene
AT5G34960.1		transposable_element_gene
AT5G34965.1		transposable_element_gene
AT5G34970.1		transposable_element_gene
AT5G34980.1		transposable_element_gene
AT5G34990.1		transposable_element_gene
AT5G34985.1		transposable_element_gene
AT5G35000.1		transposable_element_gene
AT5G35010.1		transposable_element_gene
AT5G35020.1		transposable_element_gene
AT5G35021.1		transposable_element_gene
AT5G35023.1		transposable_element_gene
AT5G35025.1		transposable_element_gene
AT5G35030.1		transposable_element_gene
AT5G35040.1		transposable_element_gene
AT5G35045.1		transposable_element_gene
AT5G35046.1		transposable_element_gene
AT5G35048.1		transposable_element_gene
AT5G35050.1	Other (1.0)	hypothetical protein
AT5G35052.1		transposable_element_gene
AT5G35057.1		transposable_element_gene
AT5G35061.1		transposable_element_gene
AT5G35065.1		transposable_element_gene
AT5G35067.1	Other (1.0)	hypothetical protein
AT5g34945.1		pseudogene
AT5G35069.1	Other (0.6), Mitochondrion (0.3)	a small protein and has either evidence of transcription or purifying selection
AT5G35070.2		pseudogene of hypothetical protein
AT5G35073.1		transposable_element_gene
AT5G35076.1		transposable_element_gene
AT5G35080.1	Endoplasmic reticulum	ATOS9/OS9; a protein involved in the endoplasmic reticulum-associated degradation of glycoproteins
AT5G35090.1	Other (1.0)	hypothetical protein
AT5G35100.1<br>AT5G35100.2<br>AT5G35100.3	Chloroplast thylakoid lumen	AtCYP28; Cyclophilin-like peptidyl-prolyl cis-trans isomerase family protein
AT5G35110.1	Other (1.0)	hypothetical protein
AT5G35111.1		pseudogene of Peroxidase superfamily protein
AT5G35113.1		transposable_element_gene
AT5G35116.1		transposable_element_gene
AT5G35118.1		transposable_element_gene
AT5G35120.1	Other (1.0)	MADS-box family protein
AT5G35130.1		transposable_element_gene
AT5G35140.1		transposable_element_gene
AT5G35142.1		transposable_element_gene
AT5G35145.1		transposable_element_gene
AT5G35146.1		transposable_element_gene
AT5G35148.1		transposable_element_gene

AT5G35150.1		transposable_element_gene
AT5G05175.1		Long non-coding RNA
AT5G35160.1<br>AT5G35160.2<br>AT5G35160.3<br>AT5G35160.4	Signal peptide (1.0)	TMN11; Endomembrane protein 70 protein family
AT5G35170.1<br>AT5G35170.1	Chloroplast envelope and thylakoid membrane	AMK5; adenylate kinase family protein
AT5G35180.1<br>AT5G35180.2<br>AT5G35180.3<br>AT5G35180.4	Mitochondrion	EDR2; ENHANCED DISEASE RESISTANCE protein
AT5G35190.1<br>AT5G35190.2	Signal peptide (1.0)	EXT13; proline-rich extensin-like family protein
AT5G35195.1	Other (0.9)	a defensin-like (DEFL) family protein
AT5G35200.1<br>AT5G35200.2	Other (1.0)	PICALM3; ENTH/ANTH/VHS superfamily protein
AT5G35205.1		transposable_element_gene
AT5G35207.1		transposable_element_gene
AT5G35210.1<br>AT5G35210.2	Chloroplast envelope membrane	PTM/DDP1; PHD type transcription factor with transmembrane domains/DDT-PHD protein1
AT5G35220.1	Chloroplast thylakoid membrane	EGY1/AMOS1/EVR3; ethylene-dependent gravitropism-deficient and yellow-green 1/ammonium overly sensitive 1/enchancer of variegation 3
AT5G35230.1		hypothetical protein
AT5G35300.1		hypothetical protein
AT5G35320.1	Other (1.0)	DBH-like monooxygenase
AT5G35330.1	Other (1.0)	ATMBD2/MBD2; methyl-cpg-binding domain protein 02
AT5G35338.1	Other (1.0)	MBD12; methyl-cpg-binding domain protein 12
AT5G35339.1		pseudogene
AT5G35331.1		transposable_element_gene
AT5G35332.1		transposable_element_gene
AT5G35334.1		transposable_element_gene
AT5G35336.1		transposable_element_gene
AT5G35337.1		transposable_element_gene

Supplementary Table S2. List of primers used for genomic PCR analysis of Ar50-33-pg1. Target genomic regions and expected sizes of the PCR products for wild type (W; Col) and mutant (M; Ar50-33-pg1) are shown. See Figure 6 for experimental results.

Set no.	Location	AGI code	Primer (5' -> 3')	Product size (bp)
#1	5:12656430 -12658588	AT5G33393	**[F]** TTGCCACTCTAGTTCGGACG<br>**[R]** GGTGCCAGCTCTACTTCCATA	903 (W), none (M)
#2	5:12676126-12678403	AT5G33406	**[F]** TCCTTCACCCTCTTTGAGATAGC<br>**[R]** GAGACGAGCACCTAGCGTTT	307 (W) , none (M)
#3	5:12961682-12963892	AT5G34780	**[F]** GCGGAGAAGTGGGAGAAACA<br>**[R]** CATAAAGAGCAGTGCACCCG	707 (W) , none (M)
#4	5:13108475-13111217	AT5G34850	**[F]** GGTGATAATCTCTGTGTTCTTGAGC<br>**[R]** GCTATCCCATCTCACACCAACG	820 (W) , none (M)
#5	5:13233391-13235522	AT5G34930	**[F]** GTACGAGGCGTTGCTTGATT<br>**[R]** GCTTTGCTGCTTCATCAGTG	536 (W) , none (M)
#6	5:13484606-13487546	AT5G35220	**[F]** AGAGTTGCTCTGCTGATGAG<br>**[R]** GCCTTTAGCTTATGGTGTATTGG	686 (W) , none (M)
#7	5:13523725-13525270	AT5G35330	**[F]** CGAACCGAACATTGGTGTCT<br>**[R]** TCAGCAAGTTCGTCGTTGGA	251 (W) , none (M)
#8	5:12621019-13562096	Entire deletion region	**[F]** GCGATGGCATTCTTAACACAAGAG<br>**[R]** CTCGTCCTACCATCTAACCTCG	None (W), 1,078 (M)
#9	5:12621019-12621657	5'-border of the deletion region	**[F]** (#8_F)<br>**[R]** CCTGACCACTATGTAATTCTCCGC	639 (W) , none (M)
#10	5:13561260-13562096	3'-border of the deletion region	**[F]** CGGTTTGACAACCAGGGAGA<br>**[R]** (#8_R)	837 (W) , none (M)
#11	5:24289890-24289596	AT5G60390 (EF1alpha)	**[F]** AGGCTGGTATCTCTAAGGATGGTCA<br>**[R]** GGATTTTGTCAGGGTTGTATCCG	295 (W, M)
#12	1:26084749-26085715	AT1G69390 (AtMinE1)	**[F]** TCTCTGCGACCTTAGTATCTCCTT<br>**[R]** TGAATCCCGTGAAATCAACC	967 (W, M)
